# Exploring Nutritional Quality and Environmental Impact of Canteen Menus and Meals in Institutional Settings: A Scoping Review

**DOI:** 10.3390/nu17223550

**Published:** 2025-11-13

**Authors:** Lara Chehade, Massimiliano Tucci, Cristian Del Bo’, Patrizia Riso, Daniela Martini

**Affiliations:** Division of Human Nutrition, Department of Food, Environmental and Nutritional Sciences (DeFENS), Università degli Studi di Milano, 20133 Milano, Italy

**Keywords:** catering sector, sustainable healthy diet, greenhouse gas emission, nutritional evaluation, institutional canteen

## Abstract

**Background/Objectives**: The growing prevalence of out-of-home eating is reflected in the contract catering sector, which worldwide generates billions of euros annually. Considering its large economic value and workforce, as well as the meals it offers in institutions, the sector may greatly impact human and planetary health. Thus, this scoping review aimed to analyze the availability of evidence on the nutritional quality (NQ) and environmental impact (EI) of institutional canteen menus/meals. **Methods**: The search was conducted using PubMed and Scopus databases and was limited to the period from 2013 to 2025. Quantitative articles that considered the NQ and/or EI of institutional canteen menus/meals were included. **Results**: Results revealed that most of the 107 articles included were conducted in high-income countries and in early-education centers. Additionally, most studies evaluated NQ in comparison to EI (*n* = 76 and *n* = 13, respectively), while only 18 studies evaluated both. It was also noted that interest in EI increased in recent years, with greenhouse gas emission being the most common indicator. **Conclusions**: This review contributes to identifying an imbalance in the available evidence, with substantially more data on the nutritional quality of institutional canteen menus and meals than on their environmental footprints, which are often assessed through greenhouse gas emissions while other environmental indicators remain largely overlooked. Moreover, the heterogeneity of study settings and the predominance of research conducted in developed countries limit the generalizability of current findings. Future studies should adopt a broader scope to achieve a more comprehensive understanding of the nutritional and environmental sustainability of institutional catering systems.

## 1. Introduction

Despite the term “sustainable diet” being coined almost 40 years ago, the currently acknowledged definition comes from the Food and Agriculture Organization (FAO) that described sustainable diets as “…protective and respectful of biodiversity and ecosystems, culturally acceptable, accessible, economically fair and affordable; nutritionally adequate, safe and healthy; while optimizing natural and human resources” [1]. This view was reinforced in the sixteen guiding principles crafted by the World Health Organization (WHO) and FAO [2]. The principles set the basis for “Sustainable Healthy Diets,” a concept that is a current focal point for various stakeholders, including governments, international organizations, civil society groups, the private sector, and academia [2].

Nowadays, research overall agrees that the dietary patterns most likely to confer health and environmental benefits simultaneously (i.e., sustainable healthy diets) are those that are rich in vegetables, fruits, nuts, whole grains, and legumes; have low to moderate amounts of fish, poultry, eggs, and low-fat dairy; and limit red and processed meats, added sugars, and refined carbohydrates [2,3]. Although results vary depending on the different parameters and dietary patterns compared, it has been estimated that shifting towards diets featuring these characteristics could allow a risk reduction for type 2 diabetes by 21%, cardiovascular disease by 15%, and cancer by 14% [4]. A review also estimated that the adoption of a dietary pattern mostly based on plant-based foods may decrease diet-related land use, greenhouse gas emissions, green water use, and blue water up to 76%, 49%, 21%, and 14%, respectively [5].

Despite food systems having achieved many positive results so far, including keeping up with a growing global population, offering a wide choice of foods to consumers, meliorating some forms of malnutrition, and reducing poverty, the sustainability of current production and consumption patterns also raise several major concerns [6]. The reciprocal interaction between environmental footprints, dietary patterns, and food systems may exacerbate bioavailability, nutrient composition, and food production, leading to environmental, nutritional, and economic consequences [7]. On the other hand, food systems play a critical role in shaping human health and environmental sustainability since they involve all actors and activities connected to producing, processing, distributing, consuming, and disposing of food products [8]. Particularly during distribution and consumption, the role of food catering services in the shift to more health-conscious and sustainable food systems is of interest [9]. Catering services are categorized into commercial catering and institutional catering. Commercial catering includes establishments such as restaurants, fast-food chain outlets, and cafés, while institutional catering serves canteens of institutions such as factories, hospitals, schools, and nursing homes [10,11]. In 2024, the global market size of institutional catering was valued at USD 543.14 billion, and it is projected to grow in the next years [12].

In Europe, the institutional sector has the highest purchase volume of food services, with health/welfare being responsible for 42.7% of total meals served, followed by education at 31.4%, and business and industry at 17.8% [13]. Moreover, the contract catering sector in Europe produces an annual turnover that reaches €25 billion, with a workforce of roughly 600,000 people delivering about 6 billion meals per year to employees, public servants, students, hospital patients and nursing home residents [14]. Considering the sector’s large size and economic value, data on the types and nutritional composition of the food served within catering services cannot be overlooked, as these services can shape the public’s dietary habits by improving access to and availability of sustainable healthy diets [15,16]. In this regard, institutional canteens represent a crucial setting for promoting healthy and sustainable eating habits, as they constitute a major component of the organizational food environment [17,18]. Indeed, they provide a substantial proportion of meals consumed out-of-home, particularly in schools, workplaces, hospitals, and universities, where daily eating routines are largely shaped by menu availability and food service practices [19,20,21]. Because of their structured nature and centralized procurement systems, canteens offer unique opportunities to implement and evaluate nutritional and environmental interventions at a large scale [22]. At the same time, besides influencing the dietary behaviors of individuals, institutional catering can affect the entire food supply chains and purchasing standards, thus fostering sustainability transitions within the food system [23,24,25].

Previous studies have aimed to analyze the nutritional and environmental impact of menus and meals in canteens. However, they mostly focused on either one aspect or the other, were limited to a specific country, or were conducted within a particular institution.

Given the current limited understanding of the nutritional quality and environmental impact of canteen menus and meals within the institutional catering system, the present scoping review aimed to fill this gap by mapping and analyzing existing evidence across various institutional settings. The review also examined differences across various settings and population groups.

## 2. Materials and Methods

### 2.1. Search Strategy

This scoping review was conducted following the Joanna Briggs Institute (JBI) methodology for scoping reviews. The research question was formulated using the PCC (Population, Concept, Context) framework, where the *Population* included users of institutional canteens (e.g., students, employees, patients, residents in nursing homes) worldwide; the *Concept* focused on studies evaluating the nutritional quality and/or environmental impact of canteen menus and meals; and the *Context* referred to institutional catering systems across different settings. Accordingly, the research question was: “What is the current evidence on the nutritional quality and environmental impact of meals provided in institutional canteens worldwide?” After identifying the research question, a literature search was performed using PubMed and Scopus databases [26,27]. Within the search strategy, research terms were incorporated within carefully crafted search syntaxes. These included the following terms: (canteen* OR school* OR hospital* OR nursing home OR “center* OR care home OR institut* OR cafeteria* OR food court OR universit*) AND (meal* OR lunch* OR menu OR menus) AND (diet* OR food* OR nutrition* OR environment* OR sustain*) AND (consum* OR optim* OR impact* OR intak*). Syntaxes were properly adapted for each database. Additional citations were also sought by examining reference lists of selected articles. The search was limited to the period from 2013 to 2025 in order to focus on the most recent and relevant evidence and to capture contemporary research on the nutritional quality and environmental impact of meals provided in institutional canteens. The search was conducted in November 2023 and updated in October 2025 to ensure the review incorporates the latest available evidence.

The literature identification process was conducted in compliance with the Preferred Reporting Items for Systematic Reviews and Meta-Analyses (PRISMA) statement [28] and the PRISMA checklist can be found in Supplementary Table S1. Since scoping reviews are not currently eligible for registration on PROSPERO [29], neither the present review nor the protocol was registered prior to data extraction and analysis.

### 2.2. Inclusion and Exclusion Criteria

In this review, articles were included if they were quantitative research articles that presented quantitative data considering nutritional quality and/or environmental impact of menus and meals served in institutional canteens. These articles had to be in English and peer-reviewed, and be based on studies conducted in different institutions such as schools, nursing homes, hospitals, etc. The following types of articles were excluded: non-English articles, qualitative research, articles that did not examine actual served menus and meals (e.g., examined theoretical menus), school initiatives that occurred outside of the school year (e.g., summer vacations), interventions that only involved specific diets (e.g., therapeutic diets), studies that examined menus/meals of private restaurants not associated with public institutions, studies that evaluated only one nutrient, articles without a methodology section, or studies that were published before 2013. There were no exclusion criteria based on the country of the study.

### 2.3. Study Selection and Data Extraction

First, articles were collected through electronic databases and reference lists and then exported to an Excel spreadsheet (Microsoft Excel, version 2410). Duplicates were then detected and deleted. Subsequently, articles were screened for titles and abstracts. Finally, the full text of the selected articles was screened to assess their eligibility. Two authors (L.C. and M.T.) independently reviewed the titles, abstracts, and full texts of eligible studies. When conflicts arose between the authors regarding the eligibility of certain articles, a third independent reviewer (D.M.) was consulted to reach an agreement.

The extracted data included publication year, country and setting of the study, study design, whether it involved menus or meals, meal types (breakfast, lunch, dinner, full-day, half-day), whether it involved a nutritional evaluation and/or an environmental evaluation, and the nutritional and environmental components it considered. In this review, analyses including meals collected over five or more consecutive days were considered representative of a “menu,” to distinguish them from studies assessing single meals. Conclusions regarding nutritional and environmental adequacy were also extracted. A study was considered to have evaluated adequacy only if it both mentioned and discussed the differences between its results regarding the served menus or meals and the reference recommendations.

## 3. Results and Discussion

**Figure 1:**
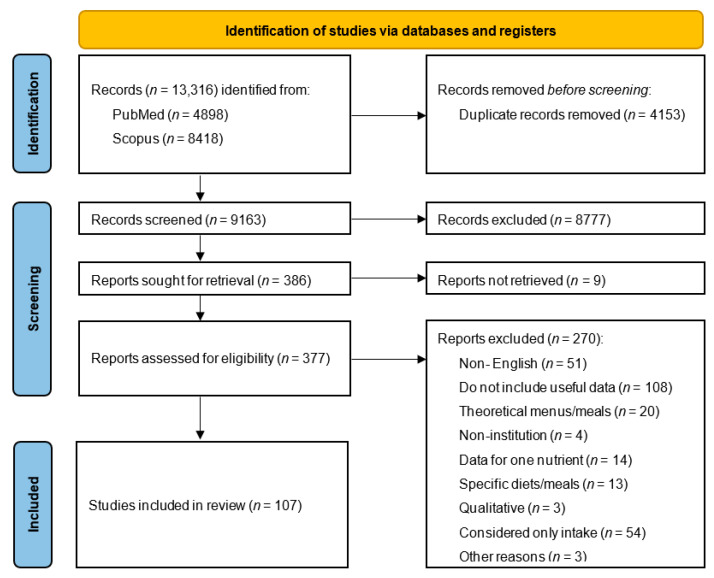
PRISMA flow chart describing the identification, screening, and selecting process of the included studies (*n* = 107).

The PRISMA flow diagram that describes the screening and selection process is shown in Figure 1. A total of 13,316 studies were retrieved from PubMed and Scopus, of which 4153 were duplicates. Out of the 9163 articles that remained, 8777 were excluded based on their title or abstract. After that, 386 articles were sought for retrieval, but 9 articles could not be accessed. Of the 377 articles that were evaluated, 270 articles were excluded according to the exclusion criteria. Overall, a total of 107 articles were included in this review.

### 3.1. Geographical Distribution

**Figure 2:**
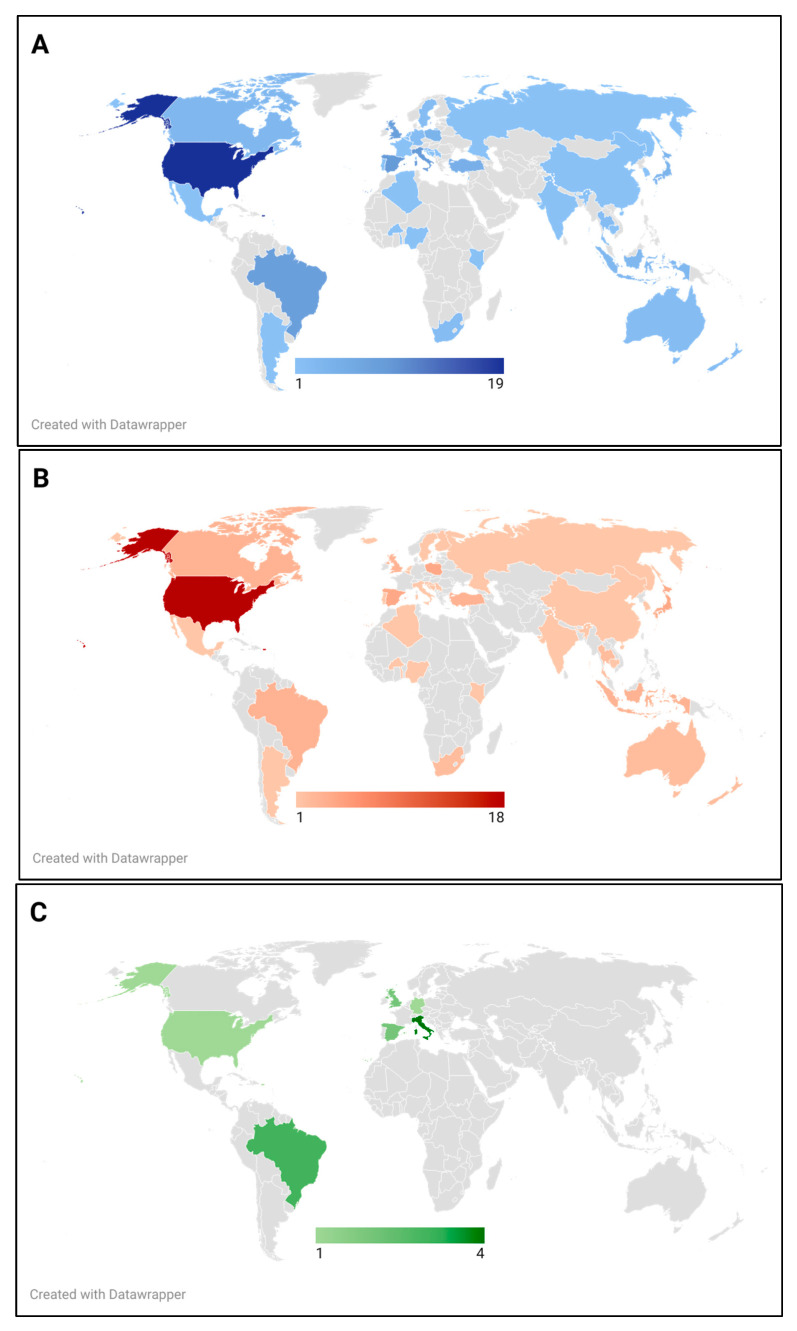
Geographical distribution of (**A**) all studies included, (**B**) studies on nutritional quality, (**C**) studies on environmental impact. Lighter colors indicate fewer available studies, while darker colors represent a greater number of studies per country.

Figure 2 depicts a geographical distribution of the included studies. They were conducted in over 37 countries, with those conducted in the United States of America being the most common (*n* = 19), followed by Italy and Spain (*n* = 9 each), and Brazil and the UK (*n* = 8 each). The analysis revealed that 93% of the studies (*n* = 100) were conducted in countries of high-income or upper-middle-income according to income classifications by the World Bank [30]. These findings are in line with a recent scoping review on environmental footprints in food services, in which all the studies included were classified as high- or upper-middle-income countries [31]. Despite the influence of globalization, which is often linked to economic, political, social, and cultural standardization across countries [32,33], food environments still differ greatly between those of high income and low-middle income [34]. Therefore, the lack of consideration for low or lower-middle income countries in research can limit the generalizability to these countries. It may also introduce biases that should be considered when developing interventions aimed at fostering the implementation of sustainable healthy menus.

### 3.2. Setting Distribution

Regarding the setting of the study, most of the studies included in this review were conducted in schools (37%) or nurseries (21%), as shown in Table 1, which presents the characteristics of the included studies. The commonality of these settings as targets of research may be due to the association between early nutrition and health. Research has shown that a poor diet during childhood may lead to obesity and NCDs in adulthood [35,36,37,38]. Therefore, ensuring that children have access to adequate and sustainable diets may promote optimal growth and development, and can reduce the risk of malnutrition later in life. It is important to note that there was a clear variation within the settings. For example, among the 38 studies that were conducted in schools, 20 were conducted in elementary/primary schools, 3 in elementary and middle schools, 3 in middle schools, and 3 in preschool and primary school. Additionally, 2 studies were conducted in boarding high schools, 3 were conducted in full school districts, 1 combined nursery, primary and secondary schools, 2 combined nursery, preschool, elementary, secondary, young adults, and adult education, and 4 did not specify the type of school. This variation across settings reflects a variation in the needs and wants of target populations as well.

### 3.3. Research Trends

Regarding the design of the studies, the most common types were observational studies (*n* = 91), of which 92% were cross-sectional and 8% were longitudinal (Table 1).

**Figure 3:**
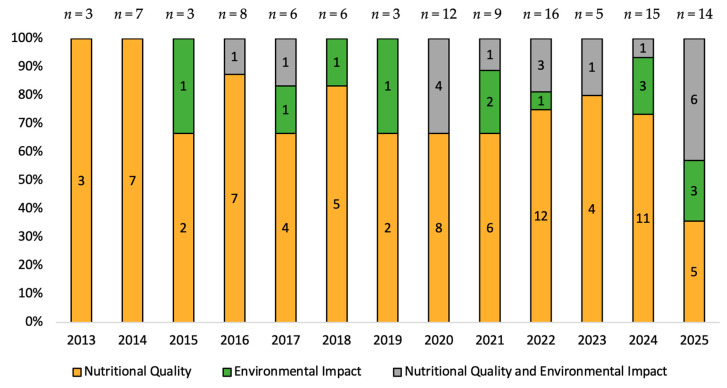
Distribution of publications across the years (2013–2025).

Out of the 107 studies included in the present review, 76 focused only on nutritional quality, 13 focused only on environmental impact, and 18 focused on both nutritional quality and environmental impact (Table 1). These results indicated a higher interest in the nutritional evaluation of menus and meals in comparison to their environmental evaluation. Moreover, as shown in Figure 3, an analysis of the publication trend between 2013 and 2025 showed a brief shift in research interest, with the focus on EI and the combination of NQ and EI being observed in the later years of the review’s specified period, despite the periodic decrease in attention noted in EI every few years (2015, 2019, and 2023). Research that focused on NQ was consistent throughout the years, with a higher percentage of NQ publications (67–100% of publications per year) being published every year in comparison to those focusing on NQ and EI combined, and only EI. The exception was 2025, where the number of studies that focused on both NQ and EI was higher than the number of those that focused solely on NQ (Figure 3). The publication pattern and the generally higher number of NQ studies across the years could have stemmed from nutrition being a more consolidated research field and the major role it has played in food policy throughout history [44,45]. Only recently, due to the alarming climate change levels and concerns about food waste, as well as efforts to ensure sustainable development, environmental footprint research has gained and is continuing to gain popularity [46,47]. In fact, the use of environmental footprints as a method of environmental sustainability assessment is relatively recent, as it was labeled a new indicator only about 10 years ago [48]. Moreover, the rise of international frameworks, such as the Paris Agreement and the United Nations Sustainable Development Goals, has emphasized reducing environmental impact and the importance of sustainable food systems [49,50]. These frameworks, along with others, may have also contributed to shifting the scientific community’s focus towards environmental sustainability.

### 3.4. Assessment of Nutritional and Environmental Components

The studies in this review varied greatly in the nutritional and environmental components they measured. Table 2 lists the different nutritional and environmental components considered within the articles. Considering the studies that assessed nutritional quality, the number of studies evaluating each nutritional component (energy, protein, carbohydrate, fat, and vitamins and minerals) was similar, ranging from 80 to 89 studies. Among the vitamins and minerals, the most considered were iron (*n* = 62), salt/sodium (*n* = 61), calcium (*n* = 61), vitamin A (*n* = 55), vitamin C (*n* = 52), and zinc (*n* = 41). Fiber was also commonly mentioned, as it appeared in 64 studies.

Concerning the different environmental components, there was a clear variation in the number of studies that evaluated them. Of the 31 studies that considered environmental evaluations (13 on EI and 18 on both NQ and EI), 10 studies assessed GHG emissions among other components, while 17 studies (55%) assessed only GHG emissions. Fewer studies considered water use (*n* = 14), land use (*n* = 2), and energy use (*n* = 3) (Table 2). The high research interest in GHG emissions noted in the present study may be due to their role in driving an unprecedented global rise in temperatures (up to 1.1 °C) since the late 19th [51,52]. Another reason for the higher focus on GHG emissions compared to other environmental indices may be that GHGs are well mixed in the atmosphere. This means that the amount of GHGs measured in the atmosphere is roughly similar all over the world, regardless of the source of the emissions [53]. In contrast, water use, energy use, and land use are more affected by region and supply, therefore greatly differ from one area to another [54,55,56,57]. This makes it harder to compare these markers globally and may contribute to them being less studied in comparison to GHG emissions. Another reason for the greater focus on GHG emissions could be the high availability of data. The SU-EATABLE LIFE Database, which compiles carbon and water footprint values of food commodities from peer-reviewed articles and grey literature, included a total of 3349 carbon footprint values from 841 publications and 937 water footprint values from 88 publications [58]. Overall, more data is available for carbon footprint than water footprint. This difference in the availability of data may have pushed researchers to conduct more research on one environmental footprint indicator than the other, which may explain the emphasis on GHG emissions. More details regarding the macronutrients, micronutrients, and indicators of environmental impact evaluated in each article, along with their units, are provided in Supplementary Table S2.

**Table 2:** Nutritional and environmental components evaluated within the included studies.

Author	Year	Nutritional Quality Components						Environmental Impact Components			
		Energy	Protein	Carbohydrate	Fat	Vitamins & Minerals	Nutritional Adequacy	GHG Emission	Water Use	Land Use	Energy Use
Adiyan et al. [41]	2025	x	x	x	x	-	-	x	x	-	-
Andersen et al. [59]	2025	x	x	x	x	x	-				
Barcina-Perez et al. [60]	2023	x	x	x	x	x	x				
Batista and Diaz [61]	2024							-	x	-	-
Biasini et al. [62]	2024	x	x	x	x	x	-				
Blondin et al. [63]	2020	x	x	x	x	x	-				
Boronowski et al. [64]	2025							x	-	-	-
Boutata et al. [65]	2024	x	x	x	x	x	x				
Buckinx et al. [66]	2017	x	x	x	x	-	x				
Bux et al. [43]	2025							x	-	-	-
Chapman et al. [67]	2022	x	x	x	x	x	x				
Cohen et al. [68]	2021	x	x	x	x	x	x				
Colombo et al. [69]	2020	x	x	x	x	x	x	x	-	-	-
Compaoré et al. [70]	2024	x	x	x	x	x	x				
Conti et al. [71]	2024							x	-	-	-
Cummings et al. [72]	2014	x	x	x	x	x	x				
Dahmani et al. [73]	2022	x	x	x	x	x	x	x	-	-	-
Deagan and Lawson [74]	2024	x	x	-	-	-	-				
De Laurentiis et al. [75]	2017							x	x	-	-
de Oliveira et al. [76]	2022	x	x	x	x	x	x				
De Seymour et al. [77]	2022	x	x	x	x	x	x				
Đermanović et al. [78]	2016	-	-	-	-	x	x				
Dixon et al. [79]	2016	x	x	x	x	x	x				
Doorduijn et al. [80]	2016	x	x	-	-	-	x				
Elinder et al. [81]	2020	x	x	x	x	x	x	x	-	-	-
Everitt et al. [82]	2020	x	-	-	-	x	x				
Farapti et al. [83]	2023	x	x	x	x	x	x				
Fitriani and Sulistiyani [84]	2024	x	x	x	x	-	x				
Flynn et al. [85]	2025							x	-	-	-
Frampton et al. [86]	2014	-	x	x	x	x	x				
Gajdoš Kljusuri et al. [87]	2016	x	x	x	x	x	x				
González-García et al. [88] ^1^	2020	x	x	x	x	x	-	x	x	-	x
González-García et al. [89]	2021							x	x	-	-
Harrison et al. [90]	2024							x	-	-	-
Hassan et al. [91]	2025	x	x	x	x	x	x				
Hatjiathanassiadou et al. [92]	2019							-	x	-	-
Holliday et al. [93]	2021	x	x	x	x	x	x				
Imamura et al. [94]	2024	x	x	-	-	x	-				
Jaworowski et al. [95]	2018	x	x	x	x	x	x				
Jindrich et al. [96]	2022	x	x	x	x	x	x				
Jiyana and Ncube [97]	2025	x	x	x	x	x	x				
Joyce et al. [98]	2018	x	x	x	x	x	x				
Joyce et al. [99]	2020	x	x	x	x	x	x				
Juniusdottir et al. [100]	2018	x	x	x	x	x	x				
Kaiser et al. [101]	2022	x	x	x	x	x	x				
Kesa and Onyenweaku [102]	2024	x	x	x	x	x	x				
Kilian et al. [103]	2021							-	x	-	-
Kluczkovski et al. [39]	2022	x	x	x	x	x	x	x	-	-	-
Knight et al. [104]	2014	x	x	x	x	x	x				
Kuruvilla et al. [105]	2021	x	x	-	x	x	x				
Lavall et al. [106]	2020	x	x	x	x	x	x				
Lavriša et al. [107]	2024	x	x	x	x	-	x				
Lazarevic et al. [108]	2014	x	x	x	x	-	x				
Leão et al. [109]	2018	x	x	x	x	x	x				
Lin et al. [110]	2024	x	x	x	x	x	x				
Lir et al. [111]	2020	x	x	x	x	x	x				
Lizuka et al. [112]	2022	x	x	x	x	x	-				
Makurat et al. [113]	2017	x	x	x	x	x	x				
Martinez-Perez et al. [114]	2025	x	x	x	x	x	x	x	-	-	-
Martins et al. [115]	2021	x	x	x	x	x	x				
Mendes et al. [116]	2025	x	x	x	x	x	x				
Menis et al. [117]	2024	x	x	x	x	-	-	x	x	-	-
Mistretta et al. [10] ^2^	2018							x	-	-	x
Mizéhoun-Adissoda et al. [118]	2022	-	-	-	-	x	x				
Moran et al. [119]	2015	x	-	-	x	x	x				
Moyano et al. [120]	2020	x	-	x	x	x	-				
Myszkowska-Ryciak and Harton [121]	2018	x	x	x	x	x	x				
Myszkowska-Ryciak and Harton [122]	2019	x	x	x	x	x	x				
Nanayakkara et al. [123]	2019	x	x	x	x	x	x				
Neelon et al. [124]	2013	x	x	x	x	-	x				
Nicklas et al. [125]	2013	x	x	x	x	-	x				
Nogueira et al. [126]	2020	x	x	x	x	x	x	-	x	-	-
Okuda et al. [127]	2024	-	-	-	-	x	-				
Ongan et al. [128]	2014	x	x	x	x	x	x				
Pepito et al. [129]	2022	x	x	x	x	x	x				
Petchoo et al. [130]	2022	x	x	x	x	-	x				
Poličnik et al. [131]	2021	x	x	x	x	x	x				
Pörtner et al. [132] ^2^	2025	x	x	x	x	x	x	x	x	x	-
Poulter et al. [133]	2024	x	x	x	x	x	x				
Rasbold et al. [134]	2016	x	x	x	x	x	x				
Retondario et al. [135]	2016	x	x	x	x	x	x				
Rodríguez-Rejón et al. [136]	2017	x	x	x	x	x	x				
Rosi et al. [42]	2022	x	x	x	x	x	x	x	x	x	-
Rossi et al. [137]	2021	x	x	x	x	x	-	x	-	-	-
Sahin and Caferoglu [138]	2022	x	x	x	x	x	x				
Sakai et al. [139]	2022	x	x	x	x	x	x				
Sato et al. [140]	2025	x	x	x	x	x	-				
Seiquer et al. [141]	2016	x	x	x	x	x	x				
Serrem et al. [142]	2020	x	x	x	x	x	x				
Shin [143]	2014	-	-	-	-	x	x				
Simon et al. [40]	2023	x	x	-	x	-	-	x	-	-	x
Sossen et al. [144]	2021	x	x	-	-	-	x				
Stanikowski et al. [145]	2020	x	x	x	x	x	x				
Takacs et al. [146] ^2^	2025	x	x	x	x	x	x	x	x	-	-
Trafalska [147]	2014	x	x	x	x	x	x				
Trang et al. [148]	2015	x	x	x	x	x	x				
Turner-McGrievy et al. [149]	2013	x	x	x	x	x	x				
Vici et al. [150]	2025	x	x	x	x	-	-	x	x	-	-
Vidal et al. [151]	2015							x	-	-	-
Volanti et al. [152]	2022							x	-	-	-
Vucea et al. [153]	2017	x	x	x	-	x	x				
Wall and Pearce [154]	2023	x	x	x	x	x	x				
Wickramasinghe et al. [155]	2016	x	-	-	-	x	x	x	-	-	-
Wickramasinghe et al. [156]	2017	x	x	x	x	x	x	x	-	-	-
Wungrath et al. [157]	2022	x	x	x	x	x	x				
Yesildemir [158]	2025	x	x	x	x	x	x	x	x	-	-
Zailani et al. [159]	2023	x	x	x	x	x	x				
Total		89	86	81	83	80	79	27	14	2	3

Articles are marked with an “x” in the column of “Nutritional Adequacy” only if they both mentioned and discussed the differences in nutritional adequacy. ^1^ The study included the recommended values for nutrients but did not provide a clear comparison. ^2^ These studies included more environmental components, including acidification (kg SO2eq), eutrophication (kg PO43−eq), and photochemical oxidation (kgC2H4eq). Note: GHG: greenhouse gas emissions.

### 3.5. Assessment of Nutritional and Environmental Adequacy

Regarding Nutritional Adequacy, 79 of the 94 articles that considered nutritional evaluations (76 focusing on NQ and 18 on both NQ and EI) performed a nutritional adequacy assessment. The articles made a comparison with the Dietary Reference Intakes, national dietary guidelines, feeding program standards, or WHO recommendations, while others used indices such as the Healthy Eating Index (HEI) and the Nutrient Rich Food (NRF) index to evaluate nutritional adequacy. Given the differences in the menus and meals evaluated, as well as the guidelines and requirements used among the different studies, nutritional adequacy evaluations varied greatly. However, despite the variation in results, there was a clear trend of menus or meals being nutritionally inadequate. In particular, this inadequacy was due to the insufficient levels of fiber and essential micronutrients such as vitamin D, vitamin E, calcium, and iron, as well as the high levels of sodium. This inadequacy may be partly explained by the limited inclusion of foods that provide fiber and micronutrients (e.g., whole grains, legumes), combined with the frequent presence of high-sodium foods, including refined bread, processed meats, and cheeses, in institutional menus. These issues may be particularly evident in institutional catering settings, where economic constraints, menu standardization, and logistical limitations often restrict the variety and frequency of nutrient-dense foods. Summaries of the nutritional adequacy conclusions of the included studies are presented in Supplementary Table S3.

Among the 31 articles that considered EI, only 5 articles compared their results against clear environmental cut-off values, which is necessary to enable a proper evaluation. Two articles compared their results with the cut-off values suggested by the SU-EATABLE LIFE project led by the Barilla Center for Food and Nutrition. In the first study, the meals showed high carbon emission and water consumption in comparison to the cut-offs [42]. In the second study, the meals offered in two out of three hospital canteens exceeded the carbon emission and the water consumption cut-offs [117]. Two other articles compared their results with the EAT–Lancet planetary boundaries [160]. Boronowsky et al. [64] showed that the calculated average of carbon emissions per meal exceeded the boundary by 3 times across all school districts they evaluated, and Conti et al. [71] reported that 99% of the menus they evaluated in a long-term care facility exceeded the carbon footprint boundary. Finally, one article compared its results with the World Wildlife Fund’s target for school lunches, showing that the baseline diet exceeded the target [81]. Different dietary guidelines and diets can greatly influence EI. For example, Gonzalez et al. summarized a list of articles that demonstrated significant variability of environmental footprint values depending on the dietary guidelines, where carbon footprint values of school lunches ranged from 1.23 to 2.35 kg CO_2_eq, and water footprint ranged from 680 to 1808 L [89]. This variation, along with the many different factors that are usually considered when calculating different environmental footprints, can only make it more difficult to establish a single universal threshold, which further complicates the assessment of environmental adequacy [161]. It was noted that, to validate their findings, most authors compared their results with baseline levels or different meal scenarios within the same study, evaluated reductions in EI in percentages, or compared results from existing research. Since the environmental impact is mostly assessed through GHG emissions given the methodological and data availability constraints discussed above, this narrow focus overlooks other critical dimensions of sustainability, such as water use, but also land occupation, and effects on biodiversity and thus limits the comprehensiveness and applicability of current findings, particularly in contexts where water scarcity or local ecosystem impacts are major concerns. Moreover, future research should also address food waste generation and management to provide a more comprehensive assessment of the environmental impact of institutional catering. In fact, food loss and waste represents a drain of the embedded environmental footprint and reducing them would directly translate into lower overall emissions and resource use.

The present scoping review has some strengths and limitations worth noting. Among the strengths, to the best of our knowledge, this is the first comprehensive review to explore available data on the nutritional quality and environmental impact of menus and meals within the institutional catering system. A previous scoping review by Guimarães et al. [31] specifically focused on the assessment of carbon and water footprints and the methodologies applied to food services, but did not consider the nutritional aspect, other environmental footprints, or the institutional catering system. In contrast to the review by Guimarães et al. [31], the present review aimed to simultaneously evaluate the availability of data for both the nutritional quality and the environmental impact of menus and meals served in institutional catering. Some findings from Guimarães et al. [31] overlap with those of the present review, particularly regarding mitigation actions such as promoting plant-based dishes and implementing educational interventions. However, our findings also highlight the need for future research to adopt an integrated nutritional and environmental impact assessment, as well as the continued need to improve the nutritional quality of institutional meals, considering the practical challenges of finding a nexus between nutritional adequacy and environmental impact reduction [162,163]. However, the harmonization of environmental impact assessments, including and expanding the use of different indicators, emerges as a key priority also from our results. Among the limitations, the search of this review was restricted to studies published in English. This language restriction may have led to the exclusion of relevant studies such as those conducted in underdeveloped or developing countries, potentially limiting the global representation of the findings. Another limitation of this review is that the menu/meal quality was assessed only in terms of energy and nutrient content. However, quality is a multidimensional concept that goes beyond nutritional composition and includes aspects related to food preparation, cooking and transport methods, meal presentation, and sensory attributes. These aspects were not considered in this review due to the limited and non-standardized nature of the available data. Finally, it is important to acknowledge that the social dimension of sustainability was not addressed in this review. Social sustainability encompasses aspects such as equity, cultural acceptability, fair working conditions, and accessibility of healthy and sustainable meals. Considering these factors in future research would allow for a more holistic assessment of institutional catering systems and support the development of policies that promote not only nutritional and environmental sustainability, but also social equity.

## 4. Conclusions

The findings of this review indicate that the availability of data is higher for the evaluation of nutritional quality than for environmental footprints, with a consistent trend of inadequacy for specific micronutrients, including vitamin D, vitamin E, calcium, iron, and high sodium content. Environmental analyses remain limited, mostly focusing on GHG emissions, while other critical dimensions such as water use, land use, and energy use are largely unexplored. Broader geographical coverage, particularly through studies conducted in low- and middle-income countries, is also needed to improve generalizability and reflect diverse food systems and resource constraints.

Thus, future research should aim to adopt standardized and comparable indicators for environmental impact assessment, allowing consistent cross-study comparisons and meta-analyses. In this regard, developing a shared repository of studies assessing both nutritional and environmental parameters in institutional meal settings would support data harmonization and comparison. The integration of nutritional and environmental dimensions should become a common practice in institutional menu planning, encouraging nutritionists and canteen managers to jointly design meals that are both nutritionally adequate and environmentally sustainable. In addition to nutritional and environmental dimensions, future research should also consider the social aspects of sustainability, such as equity, cultural acceptability, and accessibility, to provide a truly comprehensive evaluation of institutional catering systems.

This comprehensive approach will provide a more holistic understanding of the nutritional quality and environmental impact of canteen menus in an institutional context, which will facilitate the identification of gaps that need addressing, and foster progress toward sustainable and healthy diets at a global level.

## Figures and Tables

**Table 1:** Characteristics of included studies.

	All Articles (*n* = 107)
**Study Design**	
Observational	91 (85%)
Cross-sectional	84
Longitudinal	7
Interventional	10 (9%)
Uncontrolled	7
Controlled	3
Modeling	4 (4%)
Other	2 (2%)
**Nutritional vs. Environmental**	
Nutritional	76 (71%)
Environmental	13 (12%)
Both	18 (17%)
**Study Setting ^1^**	
Nursery/Preschool	22 (21%)
School	38 (37%)
University	10 (10%)
Nursing Home	9 (9%)
Hospital/community health	16 (16%)
Worksite	5 (5%)
Prison	3 (3%)
**Menu vs. Meal**	
Menus	92 (86%)
Meals	15 (14%)
**Type of Menus/Meals**	
Lunch	58 (54%)
Dinner	1 (1%)
Half day (breakfast and lunch) ^2^	12 (11%)
Full day (breakfast, lunch, and dinner)	29 (27%)
Unspecified	7 (7%)

^1^ Three studies were conducted in both schools and nurseries/preschools combined, while 1 study analyzed data from preschool, hospital, worksite, nursing home, and prison [10,39,40,41]. These 4 studies were not counted within the categories, which is why the total shown in this section of the table is 103 studies instead of 107. ^2^ Two studies included lunch and dinner, instead of breakfast and lunch [42,43].

## Data Availability

The original contributions presented in this study are included in the article/Supplementary Materials. Further inquiries can be directed to the corresponding author.

## Supplementary Materials

The following supporting information can be downloaded at: https://www.mdpi.com/article/10.3390/nu17223550/s1, Table S1: PRISMA 2020 checklist; Table S2: Characteristics of the studies evaluating nutritional quality and/or environmental impact of menus or meals in institutional settings; Table S3: Summary of Nutritional Adequacy Conclusions of Menus/Meals served in Included Studies.
