# Supplementary material for: Exploring Nutritional Quality and Environmental Impact of Canteen Menus and Meals in Institutional Settings: A Scoping Review
Source: Nutrients. 2025 Nov 13;17(22):3550. doi: 10.3390/nu17223550 (PMC12655278; doi:10.3390/nu17223550)
Supplement: Supplementary file 1 [file nutrients-17-03550-s001.zip › nutrients-3898860-supplementary.pdf]

Table S1. PRISMA 2020 checklist.

Section and Topic	Item #	Checklist item	Location where item is reported
**TITLE**			
Title	1	Identify the report as a systematic review. (Scoping)	Line 2
**ABSTRACT**			
Abstract	2	See the PRISMA 2020 for Abstracts checklist.	Line 9
**INTRODUCTION**			
Rationale	3	Describe the rationale for the review in the context of existing knowledge.	Lines 58 -94
Objectives	4	Provide an explicit statement of the objective(s) or question(s) the review addresses.	Lines 95-103
**METHODS**			
Eligibility criteria	5	Specify the inclusion and exclusion criteria for the review and how studies were grouped for the syntheses.	Line 127
Information sources	6	Specify all databases, registers, websites, organisations, reference lists and other sources searched or consulted to identify studies. Specify the date when each source was last searched or consulted.	Line 105
Search strategy	7	Present the full search strategies for all databases, registers and websites, including any filters and limits used.	Lines 105-126
Selection process	8	Specify the methods used to decide whether a study met the inclusion criteria of the review, including how many reviewers screened each record and each report retrieved, whether they worked independently, and if applicable, details of automation tools used in the process.	Lines 140-147
Data collection process	9	Specify the methods used to collect data from reports, including how many reviewers collected data from each report, whether they worked independently, any processes for obtaining or confirming data from study investigators, and if applicable, details of automation tools used in the process.	Lines 140-157
Data items	10a	List and define all outcomes for which data were sought. Specify whether all results that were compatible with each outcome domain in each study were sought (e.g. for all measures, time points, analyses), and if not, the methods used to decide which results to collect.	Lines 148-157
	10b	List and define all other variables for which data were sought (e.g. participant and intervention characteristics, funding sources). Describe any assumptions made about any missing or unclear information.	NA
Study risk of bias assessment	11	Specify the methods used to assess risk of bias in the included studies, including details of the tool(s) used, how many reviewers assessed each study and whether they worked independently, and if applicable, details of automation tools used in the process.	NA
Effect measures	12	Specify for each outcome the effect measure(s) (e.g. risk ratio, mean difference) used in the synthesis or presentation of results.	NA
Synthesis methods	13a	Describe the processes used to decide which studies were eligible for each synthesis (e.g. tabulating the study intervention characteristics and comparing against the planned groups for each synthesis (item #5)).	NA
	13b	Describe any methods required to prepare the data for presentation or synthesis, such as handling of missing summary statistics, or data conversions.	NA

Section and Topic	Item #	Checklist item	Location where item is reported
	13c	Describe any methods used to tabulate or visually display results of individual studies and syntheses.	NA
	13d	Describe any methods used to synthesize results and provide a rationale for the choice(s). If meta-analysis was performed, describe the model(s), method(s) to identify the presence and extent of statistical heterogeneity, and software package(s) used.	NA
	13e	Describe any methods used to explore possible causes of heterogeneity among study results (e.g. subgroup analysis, meta-regression).	NA
	13f	Describe any sensitivity analyses conducted to assess robustness of the synthesized results.	NA
Reporting bias assessment	14	Describe any methods used to assess risk of bias due to missing results in a synthesis (arising from reporting biases).	NA
Certainty assessment	15	Describe any methods used to assess certainty (or confidence) in the body of evidence for an outcome.	NA
**RESULTS**			
Study selection	16a	Describe the results of the search and selection process, from the number of records identified in the search to the number of studies included in the review, ideally using a flow diagram.	Lines 159-169
	16b	Cite studies that might appear to meet the inclusion criteria, but which were excluded, and explain why they were excluded.	Line 168
Study characteristics	17	Cite each included study and present its characteristics.	Table 2 and Supplementary Table 2
Risk of bias in studies	18	Present assessments of risk of bias for each included study.	NA
Results of individual studies	19	For all outcomes, present, for each study: (a) summary statistics for each group (where appropriate) and (b) an effect estimate and its precision (e.g. confidence/credible interval), ideally using structured tables or plots.	NA
Results of syntheses	20a	For each synthesis, briefly summarise the characteristics and risk of bias among contributing studies.	NA
	20b	Present results of all statistical syntheses conducted. If meta-analysis was done, present for each the summary estimate and its precision (e.g. confidence/credible interval) and measures of statistical heterogeneity. If comparing groups, describe the direction of the effect.	NA
	20c	Present results of all investigations of possible causes of heterogeneity among study results.	NA
	20d	Present results of all sensitivity analyses conducted to assess the robustness of the synthesized results.	NA
Reporting biases	21	Present assessments of risk of bias due to missing results (arising from reporting biases) for each synthesis assessed.	NA
Certainty of evidence	22	Present assessments of certainty (or confidence) in the body of evidence for each outcome assessed.	NA
**DISCUSSION**			
Discussion	23a	Provide a general interpretation of the results in the context of other evidence.	Lines 176-184,

Section and Topic	Item #	Checklist item	Location where item is reported
			198-210, 236-247, 264-282, 304-309, 329-340
	23b	Discuss any limitations of the evidence included in the review.	Line 357
	23c	Discuss any limitations of the review processes used.	NA
	23d	Discuss implications of the results for practice, policy, and future research.	Lines 373-382
**OTHER INFORMATION**			
Registration and protocol	24a	Provide registration information for the review, including register name and registration number, or state that the review was not registered.	Statement was added (Lines 122-126)
	24b	Indicate where the review protocol can be accessed, or state that a protocol was not prepared.	Statement was added (Lines 122-126)
	24c	Describe and explain any amendments to information provided at registration or in the protocol.	NA
Support	25	Describe sources of financial or non-financial support for the review, and the role of the funders or sponsors in the review.	Line 398
Competing interests	26	Declare any competing interests of review authors.	Line 407
Availability of data, code and other materials	27	Report which of the following are publicly available and where they can be found: template data collection forms; data extracted from included studies; data used for all analyses; analytic code; any other materials used in the review.	Line 405

Table S2. Characteristics of the studies evaluating nutritional quality and/or environmental impact of menus or meals in institutional settings.

Author	Year	Country	Setting	Study design	Nutritional Components	Environmental Components	Meal or Menu	Menu/ Meal Type
Adiyan et al. [41]	2025	Turkey	Preschool, Hospitals, Workplace, Nursing home, Prison	Cross-sectional	Energy (kcal), CHO (g), fat (g), protein (g)	Carbon footprint (kg/CO2 eq), water footprint (m3/ton)	Menu	Lunch
Andersen et al. [59]	2025	US	University	Cross-sectional	Energy (kcal), total CHO (g), sugar (g), fiber (g), total fat (g), SFA (g), protein (g), NA (mg)		Menu	Unspecified
Barcina-Perez et al. [60]	2023	Spain	Hospital	Cross-sectional	Energy (kcal), CHO (g), protein (g), fat (g), fiber (g), vit. B1 (mg), vit. B2 (mg), vit. B3 (mg), vit. B6 (mg), folate (µg), vit. B12 (µg), vit. A (µg), vit. C (mg), vit. E (mg), vit. D (IU), vit. K (µg), Ca (mg), Fe (mg), Mg (mg), P (mg), K (mg), Na (mg), Zn (mg), Se (µg), Cu (µg)		Menu	Full day
Batista and Dias [61]	2024	Brazil	University restaurant	Cross-sectional		Water footprint (L)	Menu	Lunch

Biasini et al. [62]	2024	Italy	Primary Schools	Cross-sectional	Energy (kcal), CHO (g), soluble sugar (g), dietary fiber (g), fat (g), SFA (g), protein (g), cholesterol (mg), vit. A (mg RE), vit. B1 (mg), vit. B2 (mg), vit. B3 (mg), vit. B6 (mg), folate (µg), vit. B12 (µg), vit. C (mg), vit. D (µg), Na (mg), K (mg), Ca (mg), P (mg), Mg (mg), Fe (mg), Zn (mg), Cu (mg).		Menu	Lunch
Blondin et al. [63]	2020	USA	Urban School District	Longitudinal	Energy (kcal), total fat (g), SFA (g), cholesterol (mg), Na (mg), total CHO (g), fiber (g), sugar (g), protein (g), vit. A (IU), vit. C (mg), Ca (mg), Fe (mg)		Menu	Lunch
Boronowsky et al. [64]	2025	USA	Primary schools	Modelling		Carbon Emission (g CO2eq)	Menu	Lunch
Boutata et al. [65]	2024	Algeria	Hospitals	Cross-sectional	Energy (kJ), protein (g), fat (g), CHO (g), fiber (g), Fe (g), Ca (mg), vit. C (mg)		Menu	Full day
Buckinx et al. [66]	2017	Belgium	Nursing homes	Cross-sectional	Energy (kcal), protein (g), protein (g/kg/d), fat (g), CHO (g)		Menu	Full day
Bux et al. [43]	2025	Italy	Hospitals and hospital wards	Cross-sectional		Carbon footprint (kgCO2eq)	Menu	Half day
Chapman et al. [67]	2022	USA	Elementary school districts	Longitudinal	Energy (kcal), total fat (g), SFA (g), Na (mg), total sugar (g), fiber (g)		Menu	Half day
Cohen et al. [68]	2021	USA	Elementary/K-8 schools	Cross-sectional	Energy (kcal), total fat (g), SFA (g), sugar (g), Na (mg)		Menu	Lunch

Colombo et al. [69]	2020	Sweden	Primary schools	Uncontrolled interventional	Energy, CHO, fat, protein, fiber, SFA, MUFA, PUFA, vit. A, vit. D, vit. E, vit. B1, vit. B2, vit. C, vit. B3, vit. B6, vit. B12, Folate, P, I, Fe, Ca, K, Mg, salt, Se, Zn *(data presented as percentages of Recommended intake)	GHGE (gCO2eq)	Menu	Lunch
Compaoré et al. [70]	2024	Burkina Faso	Primary schools	Cross-sectional	Energy (kcal), protein (g), fat (g), CHO (g), Ca (mg), Mg (mg), Fe (mg), P (mg), vit. A (µg E.R), vit. C (mg)		Meal	Lunch
Conti et al. [71]	2024	Italy	Nursing home	Modelling		Carbon footprint (kgCO2eq)	Menu	Full day
Cummings et al. [72]	2014	USA	School districts	Longitudinal	Energy (kcal), protein (g), fiber (g), total fat (g), SFA (g), sugar (g), Na (mg)		Menu	Half day
Dahmani et al. [73]	2022	France	Primary school	Cross-sectional	Energy (kcal), protein (g), fiber (g), vit. B1 (mg), vit. B2 (mg), vit. B3 (mg), vit. B6 (mg), vit. B9 (µg), vit. B12 (µg), vit. C (mg), vit. D (µg), vit. E (mg), vit. A (µg), Ca (mg), K (mg), Fe (mg), Mg (mg), Zn (mg), Cu (mg), I (µg), Se (µg), LA (g), ALA (g), DHA (mg), SFA (g), salt (g), total sugars (g)	GHGE (kgCO2eq)	Menu	Lunch
Deagan and Lawson [74]	2024	Australia	Hospital	Uncontrolled interventional	Energy (kJ), protein (g)		Menu	Full day

De Laurentiis et al. [75]	2017	UK	Primary schools	Cross-sectional		Carbon footprint (kgCO2e), water footprint (m^3)	Menu	Lunch
de Oliveira et al. [76]	2022	Brazil	Municipal Child Daycare Centers	Cross-sectional	Energy (kJ), energy (kcal), protein (g/day), CHO (g/day), fat (g/day), fiber (g), vit. C (mg), vit. A (mcg), Ca (mg), Mg (mg), Fe (mg), Zn (mg)		Menu	Full day
De Seymour et al. [77]	2022	New Zealand	Government-funded school lunch program	Cross-sectional	Energy (kJ), protein (g), total fat (g), SFA (g), CHO (g), fiber (g), vit. B1 (mg), vit. B2 (mg), vit. B3 (mg), vit. C (mg), vit. B6 (mg), vit. B12 (μg), folate (μg), vit. A equivalents (μg), Na (mg), Mg (mg), Ca (mg), Fe (mg), Zn (mg), Se (μg), I (μg)		Menu	Lunch
Đermanović et al. [78]	2016	Bosnia	Preschool	Cross-sectional	Fe, Mg, Zn, Cu, Ca (mg/daily meal)		Meal	Half day
Dixon et al. [79]	2016	USA	Child-care centers	Cross-sectional	Energy (kcal), energy (kJ), CHO (g), fiber (g), fat (g), protein (g), vit. B1 (mg), vit. B2 (mg), vit. B3 (mg), vit. B6 (mg), Folate (μg), vit. B12 (μg), vit. A (RE), vit. C (mg), vit. E (mg α), vit. D (μg), vit. K (μg), Ca (mg), Fe (mg), Mg (mg), Zn (mg)		Meal	Unspecified
Doorduijn et al. [80]	2016	Netherlands	Hospital	Longitudinal	Energy (kcal), protein (g/kg BW)		Menu	Full day

Elinder et al. [81]	2020	Sweden	Primary schools	Uncontrolled interventional	Energy (kcal), CHO (%E), fat (%E), protein (%E), fiber (%E), SFA (%E), MUFA (%E), PUFA (%E), vit. A (μg), vit. D (μg), vit. E (mg), vit. B1 (mg), vit. B2 (mg), vit. C (mg), vit. B3 (mg), vit. B6 (mg), vit. B12 (μg), folate (μg), P (mg), I (μg), Fe (mg), Ca (mg), K (mg), Mg (mg), salt (g), Se (μg), Zn (mg), added sugars (E%),	GHGE (gCO2eq)	Menu	Lunch
Everitt et al. [82]	2020	Canada	Elementary school	Cross-sectional	Energy (kcal), Na (mg)		Meal	Lunch
Farapti et al. [83]	2023	Indonesia	Nursing home	Cross-sectional	Energy (kcal), CHO (g), fat (g), protein (g), fiber (g), Na (mg), K (mg), Ca (mg)		Menus	Full day
Fitriani and Sulistiyani [84]	2024	Indonesia	Primary School	Cross-sectional	Energy (kcal), protein(g), fat (g), CHO (g)		Menus	Lunch
Flynn et al. [85]	2025	UK	University residence	Uncontrolled interventional		Carbon footprint (g CO2e)	Menu	Dinner
Frampton et al. [86]	2014	USA	Child-care centers	Cross-sectional	CHO (g), Protein (g), % fat, fiber (g), Ca (mg), Fe (mg), Mg (mg), Na (mg), Zn (mg), vit. A (μg), vit. C (mg), vit. D (μg), vit. E (mg), folate (μg)		Menu	Lunch

Gajdoš Kljusuri et al. [87]	2016	Croatia	Boarding School	Cross-sectional	Energy (kJ), fats (g), CHO (g), protein (g), total fiber (g), SFA (g), MUFA (g), PUFA (g), TFA (g), cholesterol (mg), Na (mg), K (mg), Ca (mg), Mg (mg), P (mg), Fe (mg), Cu (mg), Zn (mg), Mn (mg), Se (μg), I (μg), carotene (μg), retinol (μg), vit. D (μg), vit. E (mg), vit. B1 (mg), vit. B2 (mg), vit. B3 (mg), vit. B6 (mg), vit. B12 (μg), folate (μg), vit. B5 (mg), biotin (μg), vit. C (mg), vit. K (μg), b-carotene (μg)		Menu	Full day
González-García et al. [88]	2020	Spain	Public nursery	Cross-sectional	Fiber (g), protein (g), CHO (g), fat (g), Ca (mg), energy (kcal)	Energy (Wh), GHGE (gCO2eq), WF (L)*	Menu	Lunch
González-García et al. [89]	2021	Spain	Public nursery	Cross-sectional		Water Footprint (L), GHGE (kgCO2eq)	Menu	Lunch
Harrison et al. [90]	2024	Germany	Hospital	Controlled interventional		GHGE (kg CO2eq)	Menu	Lunch

Hassan et al. [91]	2025	Lebanon	Hospitals	Cross-sectional	Energy (kcal), total fat (g/day), cholesterol (mg/day), SFA (g/day), MUFA (g/day), PUFA (g/day), LA (g/day), ALA (g/day), trans fat (g/day), CHO (g/day), total sugar (g/day), vit. C (mg/day), vit. B1 (mg/day), vit. B2 (mg/day), vit. B3 (mg/day), vit. B6 (mg/day), folate (µg/day), vit. B12 (µg.day), biotin (µg/day), vit. B5 (mg/day), Ca (mg/day), P (mg/day), Mg (mg/day), vit. D (IU/day), vit. A (RAE µg/day), vit. E (mg/day), vit. K (µg/day), Fe (mg/day), Zn (mg/day), Na (g/day), K (g/day), I (µg/day), Cu (µg/day), Mn (mg/day), Se (µg/day), F (mg/day), Cr (µg/day), Mo (µg/day)		Menu	Full day
Hatjiathanassiadou et al. [92]	2019	Brazil	Public university restaurant	Cross-sectional		Water footprint (L/day)	Menu	Lunch
Holliday et al. [93]	2021	USA	Prison	Cross-sectional	Energy (kcal), protein (%E), CHO (%E), fat (%E), SFA (%E), fiber (g), vit. A (µg), vit. B1 (µg), vit. B2 (mg), vit. B3 (mg), vit. B5 (mg), vit. B6 (mg), folate (µg), vit. B12 (mg), vit. C (mg), vit. D (µg), vit. E (mg), vit. K (µg), Ca (mg), Cu (µg), I (mg), Fe (mg), Mg (mg), Mn (µg), P (mg), K (mg), Se (µg), Na (mg), Zn (µg)		Menu	Full day

Imamura et al. [94]	2024	Japan	University cooperation association	Cross-sectional	Energy (g), protein (g), salt (g)		Meals	Lunch
Jaworowski et al. [95]	2018	UK	Hospital Staff Canteens	Cross-sectional	Energy (kcal/portion), protein (g/portion), total fat (g/portion), SFA (g/portion), CHO (g/portion), fiber (g/portion), salt (g/portion)		Meal	Lunch
Jindrich et al. [96]	2022	USA	Childcare centers	Cross-sectional	Energy (kcal), protein (g), CHO (g), fat (g), SFA (g), MUFA (g), PUFA (g), fiber (g), folate DFE (µg), vit. A RAE (µg), Ca (mg), vit. B12 (µg), Zn (mg), K (mg), Fe (mg), Na (mg), choline (mg)		Menu	Lunch
Jiyana and Ncube [97]	2025	South Africa	Hospitals	Cross-sectional	Energy (kJ), protein (g), CHO (g), fat (g), Fe (mg), folate (µg), vit. A (µg), vit. B6 (µg)		Menu	Full day
Joyce et al. [98]	2018	USA	Local school district	Cross-sectional	Energy (kcal), protein (g), CHO (g), SFA (g), TFA (g), Na (mg), fiber (g), total fat (g), MUFA (g), PUFA (g), cholesterol (mg), sugar (g), vit. A (IU), vit. C (mg), vit. D (IU), folate (mcg), vit. B12 (mcg), Fe (mg), Ca (mg), P (mg), Mg (mg), Zn (mg)		Menu	Lunch

Joyce et al. [99]	2020	USA	Middle schools	Cross-sectional	Energy (kcal), protein (g), CHO (g), total fiber (g), sugar (g), added sugar (g), total fat (g), SFA (g), MUFA (g), PUFA (g), TFA (g), cholesterol (mg), vit. A (IU), vit. B1 (mg), vit. B2 (mg), vit. B3 (mg), vit. B6 (mg), vit. B12 (µg), biotin (µg), vit. B5 (mg), folate (µg), vit. C (mg), vit. D (IU), vit. E (mg), vit. K (µg), Ca (mg), fluoride (mg), Fe (mg), Mg (mg), P (mg), K (mg), Na (mg), Zn (mg)		Menu	Lunch
Juniusdottir et al. [100]	2018	Sweden, Finland, and Iceland	Compulsory schools	Cross-sectional	Energy (kcal), protein (g), protein (%E), fat (g), fat (%E), SFA (g), SFA (%E), CHO (g), CHO (%E), fiber (g), vit. D (µg), vit. C (mg), folate (µg), Fe (mg), Na (g)		Menu	Lunch
Kaiser et al. [101]	2022	USA	Middle school	Cross-sectional	Energy (kcal), protein (g), CHO (g), fiber (g), total sugar (g), SFA (g), Na (g), cholesterol (g), total fat (g), vit. A (µg), vit. C (mg), Ca (mg), Fe (g)		Menu	Lunch
Kesa and Onyenweaku [102]	2024	South Africa	Primary School	Cross-sectional	Energy (kcal), water (g), protein (g), fat (g), CHO (g), dietary fiber (g), minerals (g), vit. A (µg), vit. D (µg), vit. E (mg), vit. B1 (mg), vit. B2 (mg), vit. B5 (mg), vit. B6 (mg), biotin (µg), total folic acid (µg), vit. B12 (µg), vit. C (mg), Na (mg), K (mg), Ca (mg), Mg (mg), P (mg), Fe (mg), Zn (mg), Cu (mg), Mn (mg), PUFA (g)		Menu	Half day

Kilian et al. [103]	2021	Brazil	University restaurants	Cross-sectional		Water footprint (L/day)	Menu	Lunch
Kluczkovski et al. [39]	2022	Brazil	School	Controlled interventional	Energy (kcal), CHO (g/1000kcal), protein (g/1000kcal), total fat (g/1000 kcal), SFA (g/1000kcal), cholesterol (mg/1000 kcal), fiber (g/1000kcal), added sugar (g/1000kcal), Na (mg/1000kcal), Ca (mg/1000kcal), Fe (mg/1000kcal), Mg (mg/1000 kcal), Zn (mg/1000kcal), vit. A (µg/1000kcal), vit. C (mg/1000kcal), vit. B12 (µg/1000kcal)	GHGE (gCO2eq)	Menu	Half day
Knight et al. [104]	2014	USA	Child-care centers	Cross-sectional	Energy (kcal), cholesterol (mg), Na (mg), fiber (g), Fe (mg), Ca (mg), vit. A (IU), vit. C (mg), protein (g), CHO (g), total fat (g), SFA (g), TFA (g), protein (%E), CHO (%E), fat (%E)		Menu	Unspecified
Kuruvilla et al. [105]	2021	India	Community Health Centres	Cross-sectional	Energy (kcal), protein (g), Ca (mg), Fe (mg), fat (g)		Menu	Full day
Lavall et al. [106]	2020	Spain	Schools	Cross-sectional	Minerals (g/100g), protein (g/100g), fat (g/100g), CHO (g/100g), energy (kcal/100g), Na (mg/100g)		Menu	Lunch
Lavriša et al. [107]	2024	Slovenia	Nursing homes	Cross-sectional	Energy (kcal), CHO (g), total fats (g), protein (g), protein-animal source (g), protein-plant source (g), dietary fiber (g)		Menu	Full day
Lazarevic et al. [108]	2014	Serbia	Kindergartens	Longitudinal	Energy (kcal), protein (g), fats (g), CHO (g)		Menu	Half day

Leão et al. [109]	2018	Brazil	School	Cross-sectional	Energy (kcal), protein (g), fat (g), CHO (g), fiber (g), Ca (mg), Mg (mg), Fe (mg), Na (mg), Zn (mg), vit. A (μg), vit. C (mg)		Menu	Unspecified
Lin et al. [110]	2024	China	Primary and Secondary schools	Cross-sectional	Energy (kcal), protein (g), protein (%E), fat (%E), CHO (g), CHO (%E), vit. A (RAE μg), vit. B1 (mg), vit. B2 (mg), vit. C (mg), Ca (mg), Fe (mg), Zn (mg)		Menu	Lunch
Lir et al. [111]	2020	Russia	Pre-schools	Cross-sectional	Energy (kcal), protein (g), animal proteins (g), fats (g), vegetable fats (g), CHO (g), vit. B1 (mg), vit. B2 (mg), vit. C (mg), vit. A (μg RE), vit. E (mg), Ca (mg), P (mg), Mg (mg), Fe (mg)		Menu	Full day
Lizuka et al. [112]	2022	Japan	Hospital	Other	Energy (kcal), CHO (g), fat (g), protein (g), salt (g)		Meal	Lunch
Makurat et al. [113]	2017	Cambodia	Factory staff canteen	Controlled interventional	Energy (kcal), CHO (g), protein (g), fat (g), fiber (g), vit. C (mg), Fe (mg), vit. A (μg RAE), folate (μg), vit. B12 (μg)		Menu	Lunch
Martinez-Perez et al. [114]	2025	Spain	Primary schools	Cross-sectional observational	Energy (kcal), total fat (g), SFA (g), MUFA (g), PUFA (g), protein (g), total CHO (g), simple CHO (g), dietary fiber (g), Na (mg), vit. A (μg), vit. B1 (mg), vit. B2 (mg), vit. B3 (mg), vit. B6 (mg), folate (μg), vit. B12 (μg), vit. C (mg), Ca (mg), I (μg), Fe (mg), Mg (mg), Zn (mg)	GHGE (kg CO_2-eq)	Menu	Lunch

Martins et al. [115]	2021	Portug al	Public schools	Cross-sectional	Energy (kcal), protein (g), CHO (g), fat (g), fiber (g), vit. A (RAE), vit. B1 (mg), vit. B2 (mg), vit. B3 (mg), vit. B6 (mg), folate (µg), vit. B12 (µg), vit. C (mg), vit. E (mg), Ca (mg), I (µg), Fe (mg), Mg (mg), P (mg), Zn (mg)		Menu	Lunch
Mendes et al. [116]	2025	Turkey	Hospitals	Cross-sectional	Energy (kcal), CHO (g), fat (g), protein (g/d), omega 3 (g), SFA (g), dietary fiber (g/d), vit. A (µg), vit. D (µg), vit. E (mg), vit. B1 (mg), vit. B2 (mg), vit. B3 (mg), vit. B6 (mg), folate (µg), vit. B12 (µg), vit. C (mg), Na (mg), K (mg), Ca (mg), Mg (mg), P (mg), Fe (mg), Zn (mg)		Menu	Full Day
Menis et al. [117]	2024	Italy	Hospitals	Cross-sectional	Energy (kcal), protein (g), protein (%E), lipids (g), lipids (%E), CHO (g), CHO (%E), sugars (g), sugars (%E), fiber (g), fiber (%E), SFA (g), SFA (%E), MUFA (g), MUFA (%E), PUFA (g), PUFA (%E), EPA + DHA (mg)	Carbon footprint (g CO2eq), water footprint (L H2O)	Meals	Lunch

Mistretta et al. [10]	2018	Italy	School catering services	Cross-sectional		Global Energy requirement (MJprimary), Acidification (kg SO2eq), Eutrophication (kg PO43−eq), Global warming (kg CO2eq), Photochemical oxidation (kgC2H4eq)	Menu	Lunch
Mizéhoun-Adissoda et al. [118]	2022	Togo	Schools	Cross-sectional	Fe (mg), Ca (mg), vit. C (mg), vit. A (μg)		Menu	Lunch
Moran et al. [119]	2015	USA	Hospital	Uncontrolled interventional	Energy (kcal), fat (%E), SFA (%E), Na (mg), fiber (g)		Menu	Full day
Moyano et al. [120]	2020	Argentina	School	Cross-sectional	Energy (kcal), SFA (g), Na (mg), simple sugars (g), vit. A (μg), Ca (mg), Zn (mg), vit. C (mg), fiber (g), Fe (mg), vit. D (μg), folic acid (μg)		Meal	Lunch

Myszkowska-Ryciak and Harton [121]	2018	Poland	Preschools	Cross-sectional	Energy (kcal), protein (g), protein (%E), animal protein (g), vegetable protein (g), fat (g), fat (%E), SFA (%E), MUFA (%E), PUFA (%E), cholesterol (mg), CHO (g), CHO (%E), sucrose (%E), lactose (g), starch (g), fiber (g), vit. A (µg), retinol (µg), beta-carotene (µg), vit. B1 (mg), vit. B2 (mg), vit. B6 (mg), vit. B12 (µg), vit. C (mg), vit. D (µg), vit. E (mg), folate (µg), vit. B3 (mg), Ca (mg), Cu (mg), I (µg), Fe (mg), Mg (mg), P (mg), K (mg), Na (mg), Zn (mg)		Menu	Half day
Myszkowska-Ryciak and Harton [122]	2019	Poland	government-sponsored preschools	interventional uncontrolled	Energy (kcal), protein (g), protein (%E), animal protein (g), vegetable protein (g), fat (g), fat (%E), SFA (%E), MUFA (%E), PUFA (%E), cholesterol (mg), CHO (g), CHO (%E), sucrose (%E), lactose (g), starch (g), fiber (g), vit. A (µg), retinol (µg), beta-carotene (µg), vit. B1 (mg), vit. B2 (mg), vit. B6 (mg), vit. B12 (µg), vit. C (mg), vit. D (µg), vit. E (mg), folate (µg), vit. B3 (mg), Ca (mg), Cu (mg), I (µg), Fe (mg), Mg (mg), P (mg), K (mg), Na (mg), Zn (mg)		Menu	Half day

Nanayakkara et al. [123]	2019	New Zealand	Residential care facility	Cross-sectional	Energy (kJ), total protein (g), total fat (g), SFA (g), PUFA (g), MUFA (g), cholesterol (mg), total CHO (g), sugars (g), water (g), fiber (g), vit. A equivalent (μg), retinol (μg), beta carotene equivalent (μg), vit. B1 (mg), vit. B2 (mg), vit. B3 (mg), niacin equivalents (mg), vit. C (mg), vit. E (mg), vit. B6 (mg), vit. B12 (μg), folic acid (μg), folate food (μg), total dietary folate equivalent (μg), Na (mg), K (mg), Mg (mg), Ca (mg), P (mg), Fe (mg), Zn (mg), Se (μg), I (μg)		Menu	Full day
Neelon et al. [124]	2013	Mexico	Child-care centers	Cross-sectional	Energy (MJ), fat (g), fat (%E), SFA (g), SFA (%E), CHO (g), CHO (%E), protein (g), protein (%E), added sugar (g), fiber (g)		Menu	Half day
Nicklas et al. [125]	2013	USA	Child-care settings	Cross-sectional	Energy (kJ), protein (g), CHO (g), fat (g)		Meal	Lunch
Nogueira et al. [126]	2020	Brazil	Restaurants of public educational institutions	Cross-sectional	Energy (kcal), protein (g), CHO (g), fat (g), SFA (g), MUFA (g), PUFA (g), TFA (g), free sugars (g), fiber (g), Na (mg)	Water Footprint (L)	Menu	Lunch

Okuda et al. [127]	2024	Japan	Company cafeteria	Other (Pre-intervention simulation)	NaCl (g), K (mg)		Meal	Lunch
Ongan et al. [128]	2014	Turkey	Governmental elementary school	Cross-sectional	Energy (kcal), protein (g), CHO (g), fat (g), cholesterol (mg), fiber (g), carotene (μg), vit. E (mg), vit. B1 (mg), vit. B2 (mg), vit. B6 (mg), folate (μg), vit. C (mg), Na (mg), K (mg), Ca (mg), Mg (mg), P (mg), Fe (mg), Zn (mg)		Menu	Lunch
Pepito et al. [129]	2022	USA	Police training academy	Cross-sectional	Energy (kcal), CHO (g), protein (g), fat (g), vit. A (μg), vit. B1 (mg), vit. B3 (mg), vit. B6 (mg), vit. B12 (μg), vit. C (mg), vit. D (μg), vit. E (mg), Ca (mg), Fe (mg), Mg (mg), P (mg), K (mg), Se (μg), Na (mg)		Menu	Full day
Petchoo et al. [130]	2022	Thailand	Public schools	Cross-sectional	CHO (g), protein (g), fat (g), energy (kcal)		Menu	Lunch
Poličnik et al. [131]	2021	Slovenia	Primary schools	Cross-sectional	Energy (kJ), energy (kcal), protein (g), total fat (g), SFA (g), PUFA (g), MUFA (g), TFA (g), CHO (g), fiber (g), free sugar (g), salt (g)		Menu	Lunch

Pörtner et al. [132]	2025	Germany	Hospitals and nursing homes	Cross-Sectional	Energy (kcal), protein (g), total fat (g), CHO (g), fiber (g), vit. A (µg), vit. D (µg), vit. K (µg), vit. B1 (µg), vit. B2 (µg), vit. B3 (µg), vit B5 (µg), vit. B6 (µg), biotin (µg), folate (µg), vit. B12 (µg), vit. C (mg), Na (mg), K (mg), Ca (mg), Mg (mg), P (mg), Cl (mg), Fe (µg), Zn (µg), SFA (mg), MUFA (mg), LA (mg), ALA (mg), PUFA (mg), EPA & DHA (mg)	Land use (m2 × year), GHGE (kg CO2eq), Acidification (g SO2eq), Eutrophication (g PO43–eq), Scarcity-weighted water use (kL eq)	Menu	Full day
Poulter et al. [133]	2024	UK	Young Offenders Institution	Cross-sectional	Energy (kcal), CHO (g), free sugars (g), fat (g), MUFA (g), PUFA (g), SFA (g), fiber (g), protein (g), salt (g), folates (µg), vit. B1 (mg), vit. B2 (mg), vit. B3 (mg), vit. A (µg), vit. B12 (µg), vit. B6 (mg), vit. C (mg), vit. D (µg), Ca (mg), Cl (mg), Cu (mg), I (µg), Fe (mg), Mg (mg), P (mg), K (mg), Se (µg), Na (mg), Zn (mg)		Menu	Full day
Rasbold et al. [134]	2016	USA	Child-care centres	Cross-sectional	Energy (kJ), energy (kcal), protein (g), CHO (g), Fat (%), Ca (mg), Fe (mg), Mg (mg), Zn (mg), vit. A (µg), vit. E (mg), folate (µg)		Meal	Lunch
Retondario et al. [135]	2016	Brazil	Day-care centers	Cross-sectional	Energy (kJ), energy (kcal), CHO (g), Proteins (g), fat (g), fiber (g), Na (mg), Ca (mg), Fe (mg)		Menu	Full day

Rodríguez-Rejón et al. [136]	2017	Spain	Long-term care homes	Cross-sectional	Energy (kcal), protein (g), CHO (g), fiber (g), fat (g), SFA (g), MUFA (g), PUFA (g), cholesterol (mg), K (mg), Ca (mg), P (mg), Mg (mg), Fe (mg), Zn (mg), Se (µg), I (µg), Cu (µg), vit. A (µg eq), vit. D (µg), vit. E (mg eq), vit. C (mg), vit. B1 (mg), vit. B2 (mg),vit. B6 (mg), vit. B12 (µg), vit. B3 (mg), folate (µg)		Menu	Full day
Rosi et al. [42]	2022	Italy	Worksite Canteens	Longitudinal	Energy (kcal/tray), protein (g/tray), fat (g/tray), SFA (g/tray), CHO (g/tray), sugar (g/tray), fiber (g/tray), salt (g/tray)	Carbon Footprint (g CO2 eq), Water Footprint (L), Ecological Footprint (m2)	Menu	Half day*
Rossi et al. [137]	2021	Italy	Schools	Cross-sectional	Energy (kcal), fat (g), CHO (g), total sugar (g), protein (g), fiber (g), Na (mg),	GHGE (gCO2eq)	Menu	Lunch
Sahin and Caferoglu [138]	2022	Turkey	Nursing Home	Cross-sectional	Energy (kcal), protein (g), fat (g), CHO (g), fiber (g), vit. B6 (mg), folate (µg), vit. B12 (µg), vit. D (µg), Na (mg), K (mg), Ca (mg), Mg (mg), P (mg), Fe (mg), Zn (mg)		Menu	Full day
Sakai et al. [139]	2022	Indonesia	University	Cross-sectional	Energy (kcal), protein (g), fat (g), CHO (g), fiber (g), Na (mg), K (mg), Ca (mg), Mg (mg), P (mg), Fe (mg), Zn (mg), Cu (mg), Mn (mg), vit. A (µg), vit. D (µg), vit. E (mg), vit. K (µg), vit. B1 (mg), vit. B2 (mg), vit. B3 (mg), vit. B6 (mg), vit. B12 (µg), folic acid (µg), vit. C (mg), salt (g)		Menu	Lunch

Sato et al. [140]	2025	Japan	Daycare center	Cross-sectional	Energy (kcal), water (g), protein (g), fat (g), CHO (g), dietary fiber (g), Na (mg), Ca (mg), Fe (mg), vit A (μgRE), vit. C (mg)		Menu	Lunch
Seiquer et al. [141]	2016	Spain	Nursery schools	Cross-sectional	Energy (kcal), protein (g), CHO (g), fat (g), MUFA (g), PUFA (g), SFA (g), cholesterol (mg), fiber (g), vit. A (μg), vit. B1 (mg), vit. B2 (mg), vit. B6 (mg), vit. B12 (μg), vit. C (mg), vit. D (μg), vit. E (mg), vit. B3 (mg), folic acid (μg), Ca (mg), P (mg), Mg (mg), Fe (mg), Zn (mg), Na (mg), K (mg), I (μg)		Menu	Lunch
Serrem et al. [142]	2020	Kenya	Boarding high schools	Cross-sectional	Energy (kcal), minerals (g), proteins (g), fat (g), CHO (g), fiber (g), retinol (μg), vit. A (μg), beta-carotene (mg), vit. B1 (mg), vit. B2 (mg), vit. B12 (μg), vit. B6 (mg), folic acid (μg), vit. C (mg), Na (mg), K (mg), Ca (mg), Mg (mg), P (mg), Fe (mg), Zn (mg)		Menu	Unspecified
Shin [143]	2014	South Korea	Elementary schools and middle schools	Cross-sectional	Fe (μg), Zn (μg), Cu (μg), Mn (μg)		Meal	Lunch
Simon et al. [40]	2023	Spain	Public pre-school and primary education centers	Cross-sectional	Energy (kcal), protein(g), fat (g)	Cumulative Energy demand (MJ), carbon footprint – (gCO2eq)	Menu	Lunch

Sossen et al. [144]	2021	Australia	Residential care homes	Cross-sectional	Energy (kJ), protein (g)		Menu	Full-day
Stanikowski et al. [145]	2020	Poland	Prisons	Cross-sectional	Energy (kcal), protein (g), isoleucine (mg), leucine (mg), lysine (mg), methionine (mg), cysteine (mg), phenylalanine (mg), tryptophan (mg), protein (%E), fat (g), SFA (g), MUFA (g), LA (%E), ALA (%E), PUFA (g), cholesterol (mg), fat (%E), CHO (g), sucrose (g), fiber (g), CHO (%E), Na (mg), K (mg), Ca (mg), P (mg), Mg (mg), Fe (mg), Zn (mg), Cu (mg), Mn (mg), vit. A (µg), retinol (µg), vit. D (µg), vit. E (mg), vit. B1 (mg), vit. B2 (mg), vit. B3 (mg), vit. B6 (mg), vit. B12 (µg), vit. C (mg), folate (mg)		Menu	Full day
Takacs et al. [146]	2025	UK	University	Modelling	Energy (kcal), protein (g), fiber (g), SFA (g), vit. A (µg), vit. B2 (mg), folate (µg), Ca (mg), Mg (mg), K (mg), I (µg), Cu (mg), Fe (mg)	Global warming (kgCO2eq), freshwater eutrophication (kgPeq), terrestrial acidification (kgSO2eq), water depletion (m3 water eq)	Meals	Lunch

Trafalska [147]	2014	Poland	Nursery school	Cross-sectional	Energy (kcal), protein (g), fat (g), SFA (g), MUFA (g), PUFA (g), cholesterol (mg), CHO (g), saccarose (g), fiber (g), Na (mg), K (mg), Ca (mg), P (mg), Mg (mg), Zn (mg), Fe (mg), I (μg), vit. A (μg), vit. E (mg), vit. C (mg), vit. B1 (mg), vit. B2 (mg), vit. B6 (mg), vit. B3 (mg), vit. B12 (μg), vit. D (μg), folic acid (μg)		Menu	Unspecified
Trang et al. [148]	2015	Canada	Hospital	Cross-sectional	Energy (kcal), protein (g), protein (%E), protein (g/kg), fat (g), fat (%E), CHO (g), CHO (%E), Na (mg), fiber (g), cholesterol (mg), vit. C (mg), Fe (mg), Ca (mg)		Menu	Full day
Turner-McGrievy et al. [149]	2013	USA	Child-care center	Longitudinal	Energy (kcal), protein (%E), CHO (%E), fiber (g), fat (%E), SFA (%E), cholesterol (mg), Ca (mg), K (mg), Na (mg), Fe (mg), Zn (mg), vit. A (μg RAEg), vit. B6 (mg), vit. B12 (μg), vit. C (mg), vit. D (μg), vit. E (mg), vit. K (μg), folate (μg)		Menu	Half day
Vici et al. [150]	2025	Italy	Primary School	Uncontrolled interventional	Energy (kcal), CHO (g), CHO (%E), sugars (g), sugars (%E), protein (g), protein (%E), animal protein (g), animal protein (%E), vegetable protein (g), vegetable protein (%E), fat (g), fat (%E), SFA (g), SFA (%E), fiber (g), fiber (g/1000kcal)	Carbon footprint ($kgCO_2eq$/kg meal), water footprint (l/kg meal)	Menu	Lunch
Vidal et al. [151]	2015	Spain	Hospital	Cross-sectional		Carbon footprint ($kgCO_2eq$)	Menu	Full day

Volanti et al. [152]	2022	Italy	First-grade secondary school	Cross-sectional		GHGE (kgCO2eq)	Menu	Lunch
Vucea et al. [153]	2017	Canada	Long-term care homes	Cross-sectional	Energy (kcal), protein (g), CHO (g), fiber (g), vit. A (RAE), vit. B1 (mg), vit. B2 (mg), vit. B3-niacin equivalents (mg), vit. B6 (mg), vit. B12 (mcg), vit. C (mg), vit. D (mcg), vit. E (mg), folate-DFE (mcg), vit. K (mcg), vit. B5 (mg), Ca (mg), Cu (mg), Fe (mg), Mg (mg), Mn (mg), P (mg), K (mg), Se (mcg), Na (mg), Zn (mg)		Menu	Full day
Wall and Pearce [154]	2023	UK	School nurseries	Cross-sectional	Energy (kJ), energy (kcal), fat (g), fat (%E), SFA (g), SFA (%E), CHO (g), CHO (%E), dietary fiber (g), free sugars (g), free sugars (%E), protein (g), protein (%E), Fe (mg), Zn (mg), Ca (mg), vit. A (μg), vit. C (mg), Na (mg)		Menu	Lunch
Wickramasinghe et al. [155]	2016	UK	Primary schools	Cross-sectional	Energy (kcal), fiber (g), Fe (mg), Ca (mg), Zn (mg), folate (μg)	GHGE (kgCO2eq)	Menu	Lunch
Wickramasinghe et al. [156]	2017	UK	Primary Schools	Modelling	Energy (kcal), protein (g), CHO (g), non-milk extrinsic sugars (g), fat (g), SFA (g), fiber (g), Na (mg), vit. A (μg), vit. C (mg), folate (μg), Ca (mg), Fe (mg), Zn (mg)	GHGE (kgCO2eq)	Meal	Lunch
Wungrath et al. [157]	2022	Thailand	Child-care centers	Cross-sectional	Energy (kcal), protein (g), fat (g), CHO (g), fiber (g), vit. A (RAE), vit. B1 (mg), vit. B2 (mg), vit. C (mg), Fe (mg), Ca (mg), cholesterol (mg)		Menu	Lunch

Yesildemir [158]	2025	Turkey	Universities	Cross-sectional	Energy (kcal), protein (g), plant-based protein (g), animal-based protein (g), CHO (g), fat (g), SFA (g), omega 3 (g), cholesterol (mg), dietary fiber (g), vit. A (µg), vit. E (µg), vit. B1 (mg), vit. B2 (mg), vit. B6 (mg), folic acid (µg), vit. B12 (µg), vit. C (mg), Na (mg), K (mg), Ca (mg), Mg (mg), Fe (mg), Zn (mg)	Carbon footprint (CO2 eq/kg), Total (green, blue and gray) water footprint (m3/ton)	Menu	Lunch
Zailani et al. [159]	2023	Nigeria	Primary schools	Cross-sectional	CHO (g), fat (g), protein (g), fiber (g), energy (kcal), Ca (mg), K (mg), Na (mg), Fe (mg), Zn (mg), vit. C (mg), vit. A (RAE)		Menu	Unspecified

ALA: alpha-linolenic acid, BW: body weight, CHO: carbohydrate, DHA: docosahexaenoic acid, GHGE: greenhouse gas emission, IU: international unit, MUFA: monounsaturated fatty acids, PUFA: polyunsaturated fatty acids, RAE: retinol activity equivalent, RE: retinol equivalents, SFA: saturated fatty acids, TFA: trans fatty acids, WF: water footprint.

Table S3. Summary of Nutritional Adequacy Conclusions of Menus/Meals served in Included Studies.

Author	Nutritional Conclusion
Barcina-Perez et al. [60]	Energy and protein in hospital menus met the nutritional standards for the Spanish population, but insufficient provision of micronutrients such as vitamin E, vitamin D, zinc, and magnesium.
Boutata et al. [65]	Results were compared with the Dietary Reference Values. An imbalance in macronutrients distribution was noted: low fat percentage, moderate protein percentage, and a large carbohydrate percentage. Menus also did not meet the DRV for vitamin B3, B5, B9, E, and C, and iron, calcium and n-3 fatty acids.
Buckinx et al. [66]	Energy and Protein content and the nutritional value of the food served to nursing home residents did not reach the national recommended dietary allowances for a normal population.
Chapman et al. [67]	Calories and Macronutrients (Saturated fats) met USDA standards. Many meals aligned with lower sodium targets. High amount of sugar was noted in school meals.
Cohen et al. [68]	Most meals met the USDA sodium requirement (categorized as Moderate Sodium and Low Sodium).
Colombo et al. [69]	Baseline:<br>Met all dietary reference values used for planning school meals, except for saturated fatty acids and iron<br>Optimized:<br>Met all nutritional requirements
Compaoré et al. [70]	The school meals were compared with the WHO recommended nutritional requirements. The energy and CHO content of some lunches was above the recommended value. However, the content of protein was relatively low and that of lipid was high for all municipalities surveyed. The meals did not properly cover the children's micronutrient nutritional needs, except for 2 meals which notably contributed to phosphorus and vitamin A requirements.
Cummings et al. [72]	Best Practice Menu:<br>Met all requirements of the National School Lunch Program Guidelines.<br>The Typical Menu:<br>On average, the menu exceeded limits for calories, saturated fat, and sodium, but met all other National School Lunch Program Guidelines requirements
Dahmani et al. [73]	The meals provided good nutritional quality for both vegetarian and non-vegetarian meals.<br>The findings of the paper indicate that most of the RDI for many nutrients would be covered by a 2,000 kcal of the school meals, but that limit of saturated fat, salt, and total sugars would be exceeded. Coverage of calcium and alpha-linolenic acid was insufficient. Vitamin D was deficient in vegetarian meals only.

de Oliveira et al. [76]	Nutrient supply was insufficient to reach the desired Brazilian National School Feeding Program parameters.<br>Most of the nutrients did not reach the minimum recommended by PNAE for all the age groups and both periods of stay, especially Fe, Ca, vitamin A and fiber.
De Seymour et al. [77]	Meals from Ka Ora, Ka Ako menus were nutritious. Most nutrients were above 30% of the RDIs (AI for fiber). Protein and niacin were provided in levels which exceeded 100% of the daily needs of students in some age groups, but 5 nutrients were consistently below 30% of the RDIs or were low by international standards: energy, carbohydrates, iron, calcium, and iodine.<br>Sodium levels were high compared to international standards. Some menus fell below recommended levels of fiber.<br>In the years 4–8 meals, riboflavin, vitamin B6, magnesium, and selenium were marginally below a third (30%).
Đermanović et al. [78]	The daily meals in preschools do not provide adequate amounts of minerals according to the recommended dietary allowance and regulations of the Republic of Srpska.
Dixon et al. [79]	Foods and beverages provided to children met more than 50% of the Dietary Reference Intake (DRI) for energy, macronutrients, minerals and most vitamins. However, they provided less for fiber, vitamin D, and vitamin E.
Doorduijn et al. [80]	Energy did not meet the 1800 kcal a day recommendation for healthy individuals. The average protein was less than the recommended 1.0–1.25 g/kg body weight (BW) recommendation for elderly or 1.2–1.5 g/kg BW for hospitalized patients.
Elinder et al. [81]	There was no nutritional adequacy comparison done with recommendations for the baseline diet. However, the optimized diet met the EER and 30% of the DRIs for children aged 10–12 years.
Everitt et al. [82]	All meals exceeded sodium recommendations, and calories served were not sufficient as they mostly did not reach minimal daily requirements.
Farapti et al. [83]	Results were compared with the Indonesian recommended dietary allowance (RDA) for the elderly population. The energy content of the served food only met 69.03% of the recommendation, while protein met 65.62% of the recommendations. In almost all served menus, the RDA for fiber, potassium, and calcium were not met. However, sodium content of the menus exceeded the RDA.

Fitriani and Sulistiyani [84]	Most of the portion standards from the 20 menu cycles do not align with 30% of the Recommended Dietary Allowances (RDA) guidelines, known in the study as Angka Kecukup Gizi (AKG), but mainly for females. For female students, 90% of menus do not meet energy requirements, 85% for protein requirements, and 80% for fat and carbohydrate requirements, while for male students, 70% of menus meet energy requirements, 65% meet protein requirements, 80% meet those of fat, and 85% meet those of carbohydrates.
Frampton et al. [86]	Child-care center menus were insufficient in important macro and micronutrients, such as in carbohydrate, fat, dietary fiber, iron, vitamin D, and vitamin E, and contained excessive sodium. However, they provided adequate protein, magnesium, zinc, vitamin A, and vitamin C.
Gajdoš Kljusuri et al. [87]	The study revealed deficiencies in boarding school menus regarding energy and nutritional offerings in different geographic regions, and the need for better planning to improve alignment of macronutrient and micronutrient with recommendations. All menus provided sufficient energy, but the average daily intake greatly exceeded national recommendations, particularly for girls. Significant regional differences were found in sodium content. Calcium was adequate overall.
Hassan et al. [91]	The nutritional values of the food served were compared to age-specific Dietary Reference Intakes (DRIs) set by the NIH. The average energy content was lower than the estimated energy requirement, only exceeding the requirement for 19% of subjects. None of the documented macronutrient values reached 100% of the daily requirements. Results varied for micronutrients, where served foods only reached 100% of requirement for P and Ca for some of the study population groups, while they did not reach the required amounts for most of the others. Compared to the Mediterranean diet, there was low adherence to many of the food group recommendations.
Holliday et al. [93]	Menus offered excessive calories and sodium. Most macronutrients, including saturated fat, were within AMDR and majority of vitamins met or exceeded DRI levels, except for vitamins D and E, which were below recommended levels. Some menus fell below recommended levels of fiber and potassium.
Jaworowski et al. [95]	Lunch meals served in National Health Service hospital canteens provided more than 50% of recommended total fat, saturated fat, and salt for both men and women and daily energy for women. Meals were also low in fiber.
Jindrich et al. [96]	Menus did not meet the benchmark for energy, carbohydrate, Saturated fat, fiber, potassium, iron, sodium, and choline. Menus provided protein well beyond the DRI and met the benchmarks for vitamin A, vitamin D, vitamin B12, and calcium.

Jiyana and Ncube [97]	The nutrient composition was compared against the South African DRI standards. Of five public hospitals menus, the energy content of only one hospital met the RDI requirements. Meals were generally adequate in protein, while only 2 hospitals met the RDI for carbohydrates (other 2 were above and 1 was below). Only 1 hospital met the RDI for fat, while the rest exceeded it. Three out of 5 hospitals exceeded the RDI for iron, yet none of the hospitals met the folate, vitamin A, and B6 RDI.
Joyce et al. (2018) [98]	Both menus met National School Lunch Program requirements.<br>Best Practice Menu:<br>It had lower calories, saturated fat, and sodium and higher in protein, carbohydrates, fiber, vitamin A, vitamin D, phosphorus, and magnesium. The BPM had a higher Healthy Eating Index score compared to the typical menu, indicating superior overall dietary quality.<br>Typical Menu:<br>Exceeded limits for calories, saturated fat, and sodium, while providing lower amounts of other nutrients.
Joyce et al. (2020) [99]	Across the schools, the mean Healthy Eating Index score was approximately 62, which means that it needs improvement according to the USDA Center for Nutrition Policy and Promotion.<br>High-SES menus had lower added sugar and higher calcium, and more sodium than low-SES menus.
Juniusdottir et al. [100]	The guidelines and recommendations in Sweden, Finland, and Iceland were generally followed by the schools and food companies. The energy and nutrient content indicate the possibility for school meals to fulfil the recommendations and guidelines for school lunches. However, large variations were noted with in energy content as it was lower than the lower limit of the country-specific guideline in Sweden and in Finland, but not in Iceland indicating a need for standardization.
Kaiser et al. [101]	School lunches meet federal guidelines in some aspects, but students often avoid selecting healthy options. It appears that urban schools generally offered lunches that aligned with USDA nutrition standards.<br>Very few lunch selections met recommended targets for calories, fiber, vitamin C, and iron while none met recommendations for protein. No lunches meet recommendations for sugar. However, most selected lunches meet recommendations for saturated fat and cholesterol, and all meet recommendations for total fat.
Kesa and Onyenweaku [102]	The study indicated that most lunch meals did not meet the benchmark of the children's Recommended Dietary Allowances (RDAs) of 25-30%. The breakfasts provided significantly augmented the lunch meals, providing 10-20% of the RDAs. The only micronutrients that reached the RDAs target of 25-30% were vitamins B12 and A.

Kluczkovski et al. [39]	Menus had limitations in meeting the recommendations for many nutrients in specific age groups. This included insufficient content of macronutrients (except fat) and fiber, and micronutrients such as (iron, calcium, and zinc). Menus for all age groups generally exceeded saturated fats, sugar, and sodium limits.
Knight et al. [104]	Menus met energy and protein needs and provided adequate amounts of most nutrients. Most menus provided adequate carbohydrates and reached the RDA of calcium, iron, vitamin A, and vitamin C. Most menus did not reach fiber AI for children, while they went over the requirement of sodium.
Kuruvilla et al. [105]	The diets served in all the facilities were inadequate in macro- and micronutrients. All nutrients, except fat, were much lower than the recommended values.
Lavall et al. [106]	Energy supplied did not meet the recommendations on some days. Most complied with the recommendations of carbohydrates, while sodium values were exceeded. Lipid percentage varied but was higher than the recommended values for most menus. Saturated fats and PUFA contributions were at the upper limit when compared with recommendations while MUFAs were lower than the recommendations.
Lavriša et al. [107]	The menus were not balanced and did not meet the needs of the nursing home residents. The menus were high in fat, and low in quality carbohydrates, such as fiber.
Lazarevic et al. [108]	The meals were adequate. Macronutrients in total energy intake were in accordance with the national recommendations. The energy value of children's meals did not exceed the level statutory recommended.
Leão et al. [109]	The results were either very low or very high compared to the PNAE recommendations, without differentiation for each school category and their respective age groups. In menus of the nursery category, energy, macro and micronutrients were higher than the recommended values by the PNAE, except for sodium and fibers, whose percentages were lower than the reference values. For other categories, values did not meet minimum recommendations. Calcium, iron, magnesium, zinc, vitamins A and C were below the recommendations for most groups.
Lin et al. [110]	The supply of protein and fat the schools exceeded recommended standards, and the %E of macronutrients did not meet the recommendations. Regarding micronutrients, the level of calcium was low, while vitamin A content was low only in middle and non-urban primary schools.
Lir et al. [111]	Calorie, macronutrient and micronutrient contents corresponded to age-related standards at all the examined preschool children facilities. However, calcium and phosphorus quantities were not balanced well.

Makurat et al. [113]	The caloric content of lunch sets matched targeted goal (one-third of the RDI). Macronutrients and dietary fiber provided by the exemplary sets seem to be balanced and sufficient. Lunch sets contained adequate micronutrient content (adequate amounts of vitamin C, vitamin A, folate and vitamin B12, but low iron).
Martinez-Perez et al. [114]	Most menus exceeded recommended energy levels. Macronutrients were assessed using AMDRs for Spanish children, while micronutrients were evaluated using the Institute of Medicine (IOM) Dietary Reference Intakes (DRIs). The menus served at Charter schools had significantly higher energy, total fat, SFA, and carbohydrate content. Calcium and iodine levels showed major deficiencies with barely any menus meeting the recommendations. Low adequacy of dietary fiber was noted in the menus. Overall, menus were high in contents of energy, fats, proteins, and sodium, but low in carbohydrates, fiber, and certain micronutrients.
Martins et al. [115]	Majority of the meals did not meet international dietary guidelines for energy and macronutrients (below the age-specific lower limit). Some meals observed exceeded the recommended upper limit for proteins. The lunches offered also did not meet the dietary guidelines for fiber and micronutrients.
Mendes et al. [116]	The menus were compared with recommendations of the Food and Drug Administration, European Food Safety Authority, and the 2022 Türkiye Dietary Guidelines. High levels of energy, carbohydrates, fat, saturated fat, and sodium, and lower levels of calcium and fiber in the menus may increase malnutrition risk for men and women. However, regarding protein content, malnutrition risk was noted only for men. All in all, the study concluded that the menus examined are unlikely to pose a risk of malnutrition, and were to a certain point appropriately formulated.
Mizéhoun-Adissoda et al. [118]	The assessment of micronutrient intake showed low content of nutrients in comparison to recommended guidelines in the school menu. Some meals were significantly below recommended intakes for children for iron, depending on age groups. Calcium, vitamin A, and vitamin C were below the recommended intakes for the children regardless of their age.
Moran et al. [119]	At Baseline:<br>No hospital met all of the Healthy Hospital Food Initiative (HHFI) patient meal standards. Most hospitals did not meet the daily limit for percentage of calories from total fat, percentage of calories from saturated fat, and sodium. They also did not meet the fiber standard.<br>Healthy Hospital Food Initiative:<br>All menus met or exceeded the HHFI standards.

Myszkowska-Ryciak and Harton (2018) [121]	Majority of kindergartens menus were largely in line of recommendations for children aged 1–3 years, but almost a third of institutions failed to meet the recommendations of children aged 4–6 years old.<br>Preschool menus largely met the recommendations regarding the supply of nutrients, especially dietary fiber, vitamins, and selected minerals. However, the supply of vitamin D, calcium, iron, iodine, potassium, need great improvement In most kindergartens, recommendations for energy were exceeded. Energy from protein occasionally exceeded recommendations by 2- or 3-fold. In almost one-third of preschools, the energy derived from fat exceeded the recommendation. Saturated fatty acids and sodium exceeded the limit in the majority of kindergartens.
Myszkowska-Ryciak and Harton (2019) [122]	Before Education Program:<br>Children received the recommended or higher amounts of energy and macronutrients, but dietary fiber was mainly insufficient. Energy from total fat, saturated fat, and protein were high. Copper, phosphorus, sodium, and zinc met recommendations, but calcium, iron and potassium were mostly below recommendations.<br>After Education Program:<br>After education, the energy value, total carbohydrates, total fat, and percentage of energy from fat and MUFA decreased. Menus met at least 70% of the recommended intake for all vitamins except vitamin D. The nutrient density for most vitamins and minerals improved, except for vitamin D, calcium, iron, potassium, vitamin E, and iodine in some age groups.
Nanayakkara et al. [123]	The study suggested that the menus should be oversupplied to ensure adequate nutrient intake.<br>The menu did not reach recommendations for retinol, water, fiber, magnesium, calcium, phosphorous and iodine. The menu also exceeded the recommended percent of fat contribution to total energy, while percent of protein was suboptimal.
Neelon et al. [124]	Menus were in line with recommendations. Energy from fat, carbohydrates, and protein were generally in line with DRI standards. The amount of fiber provided to children was close to the DRI recommendation. The sugar content of total daily foods was below the recommended level.
Nicklas et al. [125]	In Line with recommendations. The percentage of protein, carbohydrate and fat in lunch meal did not exceed the Institute of Medicine's AMDR.
Nogueira et al. [126]	Meals served by restaurants of public educational institutions do not satisfy nutritional quality criteria. They presented excessive sodium, saturated and total fat, free sugars, trans fat.

Ongan et al. [128]	School lunch did not meet lunch energy-nutrients requirements for children, as nutrients were in excessive or inadequate amounts. School lunch contained high levels of fat, saturated fat, sodium, and lower levels of fiber and calcium.
Pepito et al. [129]	Menu met the recommended intake for most of the macronutrients and micronutrients investigated. Nutrients that did not meet adequacy were calories, carbohydrates, vitamins D, vitamin E, magnesium, and potassium.
Petchoo et al. [130]	Composition and nutritional quality of lunch meals served at schools tended to deviate from the adopted national standards. Macronutrients and energy content of the lunches provided at kindergarten exceeded the recommendation, except carbohydrates. For the elementary school, nutrients were close to the recommendations, but menus with one-dish meal did not meet the recommendation for all nutrients except protein.
Poličnik et al. [131]	School lunches had lower energy, carbohydrates, and total fat values as recommended but exceeded limits for salt, saturated fats, and polyunsaturated fats. The macronutrient energy ratios were generally favorable, but energy from carbohydrates was slightly below recommendations. While total dietary fats were below the recommended level, saturated and polyunsaturated fats were excessive, and industrial trans fats met guidelines. Protein content aligned with recommendations, but salt levels were more than twice the acceptable limit for adolescents aged 10 to 14 years.
Pörtner et al. [132]	Average daily nutrients were compared to dietary reference values issued by the German Nutritional Society. Diets in the hospitals and nursing homes were considered to be of poor quality, as reflected by Healthy Eating Index scores. Almost half of calories provided were from fat, with calories from saturated fat being substantially more than the recommended amount. Protein requirements were nearly met in hospitals, but not in nursing homes. All institutions fell short with the supply of niacin, vitamin B6, folate, pantothenic acid, potassium, magnesium, and EPA/DHA, while not all met the recommendations of biotin, vitamin C, calcium, zinc, iron, thiamine and riboflavin. Sodium and chloride consistently exceeded the dietary reference value.
Poulter et al. [133]	The 2019 and 2022 menus were compared with the UK Government dietary recommendations and EFSA tolerable upper intake Levels. In 2022, it was mainly sodium and free sugars that showed values of concern, greatly exceeding limits. Most nutrients were within safe limits though. When comparing the 2019 and 2022 menus, the 2022 menu showed improvements, such as lower fat, CHO, energy, salt, sodium, and SFA levels, and higher vitamin D and zinc.

Rasbold et al. [134]	Macronutrients and micronutrients served to preschool-aged children at child-care centers in Oklahoma were within recommended amounts or exceeded some recommendations, including protein, carbohydrates, vitamin A, zinc, magnesium, folate and calcium while being deficient in others such as vitamin E, and iron. On average, energy and total fat served were within desired ranges for these preschoolers.
Retondario et al. [135]	School feeding programs partially met the PNAE guidelines for children. For group A children, carbohydrates, proteins and Ca were generally in line with recommendations, while the program met the nutritional needs of the older children (group B) children regarding proteins and Na content and provided more than half of the DRI for lipids, fiber and iron.
Rodríguez-Rejón et al. [136]	The menus analyzed did not meet the dietary recommendations, especially regarding micronutrients. Regarding macronutrients, the protein content of regular menus met the RDA, and the amount of carbohydrates is above the minimum established, but fiber was insufficient.<br>Some micronutrients did not reach the EAR (or AI) in some or all of the menus and they included, potassium, magnesium, zinc, iodine, vitamin D, vitamin E, vitamin B3, and folate.
Rosi et al. [42]	Energy, carbohydrates, fats, and fiber were adequate, but the proportion of energy from proteins was slightly high. High salt levels were also noted.
Sahin and Caferoglu [138]	Menus were not compatible with the Turkey Dietary Guidelines.<br>The menu's protein content was below recommendations for males, and it lacked several key micronutrients (vitamin B6, folate, B12, D, potassium, calcium, magnesium, iron, and zinc) for older adults based on the Turkey Dietary Guidelines. Additionally, the menus contained high fat, sodium, and phosphorus.
Sakai et al. [139]	Nutritional values of most canteen menus were deficient in both macronutrients and micronutrients and were high in sodium content (almost twice the RDA).
Seiquer et al. [141]	Despite some imbalances, the menus were generally in line with nutritional guidelines and could serve as adequate nutritional foundations in early childhood.<br>The menus provided adequate fiber and micronutrients. However, vitamin D, calcium, and zinc levels were below recommendations, and carbohydrates contributed fewer calories than recommended. While total fats exceeded the recommendations, the PUFA/SFA ratio was in line with recommendations. Protein content was also slightly high, and cholesterol levels exceeded the suggested maximum recommendation.

Serrem et al. [142]	The meals did not provide students with adequate nutritious foods. School meals did not meet 100% of the energy needs of the students, but they met more than 75% of the nutritional requirements for macronutrients. Most micronutrient recommendations were met, except for vitamin A, vitamin C, vitamin B12, and calcium. In some cases, schools exceeded the recommended dietary fiber intake by three times.
Shin [143]	School meals provided insufficient amounts of Fe, Zn or Mn, but an excessive amount of Cu in comparison to the Korean Dietary Recommended Intakes level of microminerals.
Sossen et al. [144]	The menu provided adequate energy and protein.
Stanikowski et al. [145]	The food provided in Polish prisons and detention centers does not cover the reference standards of nutrients intake. The energy values meet the Polish recommendations for food-based energy supply for males aged 31–50 with low physical activity but they do not meet the recommendations for Polish prisons issued by of the Minister of Justice. Energy from saturated fatty acids was below the recommendations. Inadequate levels of micronutrients were noted (vitamin C, vitamin D, folate, calcium).<br>The recommended intake was most highly exceeded in the case of vitamins A, B6, and B12, sodium, manganese, copper, and phosphorus.
Takacs et al. [146]	The Nutrient Rich Food (NRF) index was used to evaluate the meals' nutrient density. According to the NRF 17.3 model, which takes into consideration the nutrients identified as of concern for UK adults, only four recipes out of 13 recipes had scores that reached at least 30% of the maximum score (>420 out of 1400).
Trafalska [147]	Menus need to be adjusted to better align with nutritional recommendations. Energy exceeded recommendations and protein content was three times higher than recommended but remained within reference values. Dietary fat content<br>was found to be rather low, with a high content of saturated fat coupled with low content of polyunsaturated fatty acids. Carbohydrates met recommendations, though sucrose consumption was excessive. Significant deficiencies were observed in vitamin D, iodine, vitamin E, and iron, while sodium, phosphorus, magnesium, zinc, and vitamins A, B2, B6, and B12 were consumed in excess.
Trang et al. [148]	Patient regular menus did not consistently meet recommendations for energy and nutrient levels. Menus varied greatly in nutritional content, and half of the standard regular menus contained less energy than the recommended levels. Even though all regular menus provided macronutrients within the AMDR, the mean protein level provided would not be sufficient to meet the protein needs of most patients who may have higher protein requirements. All regular menus met the DRI of iron, while fewer met those of calcium and vitamin C, and none met the requirements for fiber.

Turner-McGrievy et al. [149]	The menus showed a varied alignment with energy, macronutrient, and micronutrient recommendations. Most menus met the energy requirements while the meat menus exceeded energy recommendations for 3-year-olds. Only the veg-post menu met the saturated fat limit. The meat menus did not meet fiber DRIs for either age group while the vegetarian menus met the fiber DRI but did not meet the vitamin K recommendations. The menus met the Iron, zinc, and vitamin B12 requirements, but none met the potassium, vitamin E, and D requirements.
Vucea et al. [153]	There were no menus that met the DRIs for all macronutrients, micronutrients, and fiber.<br>Carbohydrates were in line with recommendations, while protein offered daily exceeded the RDA, while fiber did not meet the recommendations. Nine micronutrients (vitamins B6, vitamin D, vitamin E, vitamin K, folate, calcium, magnesium, potassium, and zinc) did not meet the DRI recommendations.
Wall and Pearce [154]	Lunches were evaluated against the food-based standards for school lunches and the voluntary food and drink guidelines for lunch in Early Years Settings (EYS), and they were more compliant with the former than the latter. Lunches were providing excess energy and protein compared to the requirements for 3 and 4 year-old children, and higher saturated fat, free sugars and Na contents of lunches compared with recommendations for younger and older age groups.
Wickramasinghe et al. (2016) [155]	Healthy meal defined by having a low level of salt, free sugars and saturated fat:<br>None of the healthy meals met standard levels of positive food elements, including fiber, iron, calcium, zinc, folate, and energy.<br>Healthy meal defined by achieving 7 or more standards from the 14 nutrient-based standards:<br>All the healthy meals met standard levels of positive food elements, including fiber, iron, calcium, zinc, folate, and energy.
Wickramasinghe et al. (2017) [156]	In the pre-school food survey group, all meals met recommendations, except for sodium, which was higher, and iron, zinc, and energy, which were lower compared to the recommendations
Wungrath et al. [157]	Child-care center lunches did not meet the nutritional guidelines required for healthy growth and development of children. Lunches were insufficient in energy, macronutrients,<br>dietary fiber, iron, vitamin B1 and calcium compared to 40% of the daily DRI, while contents of vitamin A, vitamin B2, vitamin C and the iron met recommendations.

Yesildemir [158]	The energy content of lunch menus from all universities significantly exceeded the recommended range. Regarding macronutrient proportions, those were compared to the recommendations of the Ministry of Health of the Republic of Türkiye 2022. The proportions deviated from the recommendations, with a lower protein and carbohydrate proportion and a higher fat proportion, indicating a need for nutritional adjustments in the menus. All micronutrients, including sodium, exceeded the recommendations, except for calcium which failed to reach the recommended levels.
Zailani et al. [159]	The school meals served through the National Homegrown School Feeding Program were able to provide at least one third of the recommended nutrient intake for most macronutrients and micronutrients. However, the meals did not make an adequate contribution to the recommended nutrient intake for energy, carbohydrates, fiber, potassium, and vitamin C.

AI: adequate intake, AMDR: acceptable macronutrients distribution range, BW: body weight, DRI: dietary reference intakes, EAR: estimated average requirement, EER: estimated energy requirement, PNAE: Brazilian National School Feeding Program, SES: socioeconomic status, USDA: United States Department of Agriculture.
